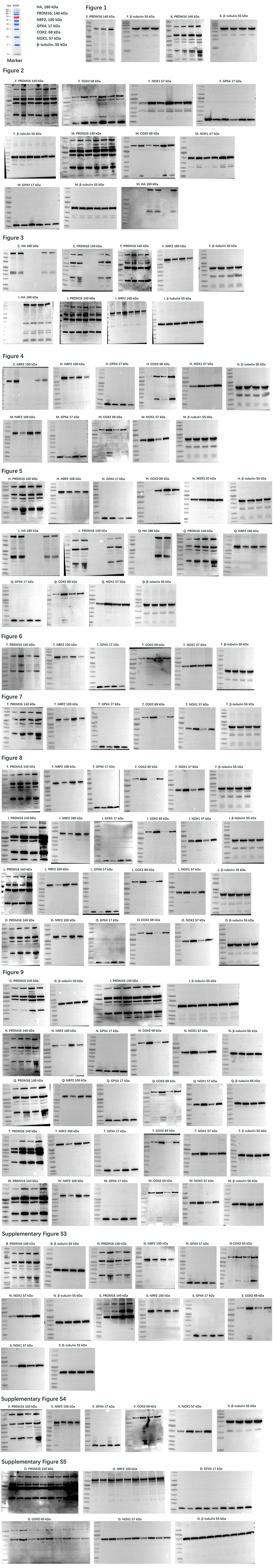# PRDM16 suppresses ferroptosis to protect against sepsis-associated acute kidney injury by targeting the NRF2/GPX4 axis

**DOI:** 10.1016/j.redox.2024.103417

**Published:** 2024-11-07

**Authors:** Qiang Zheng, Jihong Xing, Xiaozhou Li, Xianming Tang, Dongshan Zhang

**Affiliations:** a Department of Emergency, The First Hospital of Jilin University, Changchun, Jilin, China; b Department of Emergency and Critical Care Medicine, The Second Xiangya Hospital of Central South University, Changsha, Hunan, China; c Emergency Medicine and Difficult Diseases Institute, The Second Xiangya Hospital of Central South University, Changsha, Hunan, China; d Department of Nephrology, The Second Xiangya Hospital of Central South University, Changsha, Hunan, China; e Furong Laboratory, Changsha, Hunan, China

**Keywords:** AKI, Ferroptosis, GPX4, NRF2, PRDM16, Sepsis

## Abstract

Acute kidney injury (AKI) constitutes a significant public health issue. Sepsis accounts for over 50 % of AKI cases in the ICU. Recent findings from our research indicated that the PRD1-BF1-RIZ1 homeodomain protein 16 (PRDM16) inhibited the progression of diabetic kidney disease (DKD). However, its precise role and regulatory mechanism in sepsis-induced AKI remain obscure. This study reveals that lipopolysaccharide (LPS) and cecum ligation and puncture (CLP) instigated PRDM16 expression in Boston University mouse proximal tubule (BUMPT) cells and mouse kidneys, respectively. Functionally, PRDM16 curtailed LPS-induced ferroptosis. Mechanistically, PRDM16 associates with the promoter regions of nuclear factor-erythroid 2-related factor-2 (NRF2) and augments its expression, subsequently enhancing glutathione peroxidase 4 (GPX4) expression. Additionally, PRDM16 directly engages with the promoter regions of GPX4, stimulating its expression. Notably, these observations were corroborated in human renal tubular epithelial (HK-2) cells. Furthermore, the ablation of PRDM16 from kidney proximal tubules in mice inhibited NRF2 and GPX4 expression, leading to decreased glutathione (GSH)/oxidized glutathione (GSSG) ratio, increased Fe^2+^ and reactive oxygen species (ROS) production, exacerbated ferroptosis, and AKI progression. Conversely, PRDM16 knock-in exhibited the opposite effects. Ultimately, adenovirus (ADV)-PRDM16 plasmid or poly (lactide-glycolide acid) (PLGA)-encapsulated formononetin not only mitigated sepsis-induced AKI but also alleviated liver, cardiac, and lung injury. In summary, PRDM16 inhibits ferroptosis via the NRF2/GPX4 axis or GPX4 to prevent sepsis-induced multi-organ injury, including AKI. PLGA-encapsulated formononetin presents a promising therapeutic approach.

## 1 Introduction

Sepsis is a life-threatening syndrome characterized by multiple organ dysfunction due to excessive generation of reactive oxygen species (ROS) and an unregulated immune response to infection [1]. Approximately 60 % of individuals with sepsis experience acute kidney injury (AKI) [[2], [3], [4]]. The mortality rate for sepsis complicated by AKI is 2–3 times higher than that of sepsis without AKI [[5], [6], [7]]. Consequently, sepsis-associated acute kidney injury (SA-AKI) constitutes a significant global public health concern [8,9]. Current treatment strategies for sepsis primarily rely on the use of broad-spectrum antibiotics and fluid resuscitation, which are insufficient in preventing multiple organ injuries, including the kidneys [10]. Therefore, it is imperative to investigate new therapeutic targets and approaches to mitigate these consequences.

Ferroptosis, a novel form of cell death, is distinguished by the induction of lipid peroxidation, depletion of glutathione (GSH), and inactivation of glutathione peroxidase 4 (GPX4) [11]. This process is implicated in the progression of various organ diseases, including those affecting the liver, cardiovascular system, lungs, and kidneys [[12], [13], [14], [15], [16]]. Recent studies have particularly highlighted the close association between ferroptosis and SA-AKI. One investigation indicated that iridoin might offer defense against SA-AKI by inhibiting ferroptosis via the activation of the sirtuin 1 (SIRT1)/nuclear factor-erythroid 2-related factor-2 (NRF2) axis [17]. Additionally, Li J et al. demonstrated that dexmedetomidine activated the kelch-like ECH-associated protein 1 (KEAP1)/GPX4 signaling pathway, thereby inhibiting ferroptosis in SA-AKI [18]. Furthermore, maresin conjugates in tissue regeneration 1 (MCTR1) were found to inhibit ferroptosis in SA-AKI through NRF2 signaling [19]. Conversely, prostaglandin E2 (PGE2) promotes oxidative stress-induced ferroptosis in renal tubular epithelial cells by inhibiting GPX4 [20]. Despite these findings, the regulatory mechanisms of ferroptosis in sepsis remain largely unelucidated.

As a transcriptional core regulator, PRD1-BF1-RIZ1 homeodomain protein 16 (PRDM16) plays a significant role in adipocyte differentiation [21], energy metabolism [22], obesity regulation [23], and metabolic diseases [24]. Previous studies have shown that PRDM16 attenuates fibrosis, thereby suppressing the development of diabetic kidney disease (DKD) [25]. Additionally, recent research has indicated that the overproduction of ROS is a crucial feature in sepsis with or without AKI [26] and serves as a pivotal regulator of ferroptosis [27]. Another study suggested that PRDM16 protects cardiomyocytes by inhibiting ROS production [24]. Based on these findings, we hypothesized that PRDM16 could inhibit ROS production to suppress ferroptosis, thereby protecting against sepsis.

The present study demonstrated that PRDM16 suppresses ferroptosis in renal tubular epithelial cells, thereby attenuating septic AKI via upregulation of the NRF2/GPX4 axis or GPX4. The use of an adenovirus (ADV)-PRDM16 plasmid or poly (lactide-glycolide acid) (PLGA)-encapsulated formononetin significantly alleviated sepsis-associated multi-organ injury, including kidney damage, thus providing new targets and strategies for the treatment of sepsis.

## 2 Methods and materials

### 2.1 Antibodies and reagents

The PRDM16 (Catalog Number: PA5-20872) antibody was sourced from Invitrogen (Thermo Fisher Scientific Inc, Carlsbad, CA, USA). The β-tubulin antibody (Catalog Number: T0023) was secured from Affinity Bioscience (Rosemont, IL, USA). Antibodies targeting NRF2 (Catalog Number: 380773) and GPX4 (Catalog Number: 381958) were acquired from Chengdu Zen Biotechnology Co., Ltd. (China). Antibodies targeting cyclooxygenase 2 (COX2, Catalog Number: ET1610-23) and recombinant nicotinamide adenine dinucleotide phosphate oxidase 1 (NOX1, Catalog Number: ER1913-99) were acquired from Hangzhou HuaAn Biotechnology Co., Ltd. (China). The HA-Tag (Catalog Number: 3724) antibody was obtained from Cell Signaling Technology (Danvers, MA, USA). The luciferase assay kit was procured from BioVision (Milpitas, CA, USA). PRDM16 shRNA, PRDM16 plasmid, NRF2 shRNA, and NRF2 plasmid were sourced from Ribo (Guangzhou, China). Additionally, Lipofectamine 2000 was provided by Life Technologies (Carlsbad, CA, USA), and the TRIzol reagent was supplied by Accurate Biotechnology Co., Ltd (China). The PerfectStart Green qPCR SuperMix, cDNA Synthesis SuperMix, and EasyScript One-Step gDNA Removal were obtained from Transgen (China). An adenovirus (ADV)-PRDM16 plasmid was provided by Heyuan Biological (Shanghai, China). PLGA 50:50 was sourced from Meilunbio (Dalian, China). Finally, Formononetin was procured from the Guangzhou Institute of Biomedicine and Health (Guangzhou, China).

### 2.2 Cell culture and treatment

The Boston University mouse proximal tubule (BUMPT-306) cell line was established by utilizing the immortoMouse model that contains a temperature-sensitive SV40 T-antigen oncogene [28]. To achieve immortalized cell growth, the BUMPT cells were initially cultured with γ-interferon at 33 °C and then switched to γ-interferon-free medium [28]. They were kept in DMEM (Gibco, Waltham, MA, USA) comprising 10 % fetal bovine serum (FBS, 10,000 U/ml) and 1 % penicillin-streptomycin (10,000 μg/ml) at 37 °C in a 5 % CO_2_ atmosphere. The cell lines stably expressing PRDM16-RFP were established according to a prior study [25]. Under identical conditions, PRDM16-RFP stably expressed cell lines were cultured in DOX (500 ng/ml). F12 medium (Gibco, Waltham, MA, USA) was utilized to cultivate human renal tubular epithelial cells (HK-2). Subsequently, PRDM16-RFP stably expressed cell lines, BUMPT, or HK-2 cells were treated with lipopolysaccharide (LPS, Sigma-Aldrich, USA) for 24 h at concentrations of 300, 300, or 50 mg/mL, respectively. Cells were transfected utilizing PRDM16 shRNA (100 nM), PRDM16 plasmid (1 μg/ml), NRF2 shRNA (100 nM), NRF2 plasmid (1 μg/ml), or a negative control using Lipofectamine 2000 (Life Technologies, USA).

### 2.3 Animal model

Male C57 BL/6 J mice, aged between 8 and 10 weeks, were procured from Hunan SJA Laboratory Animal Co., Ltd. (Hunan, China). These mice were maintained in a regulated setting featuring a 12-h light/dark cycle with unrestricted access to food and water. All animal procedures conformed to the guiding principles sanctioned by the Animal Care Ethics Committee of Second Xiangya Hospital (NO. 2018065). Mice with proximal tubule-specific PRDM16-knockout (PT-PRDM16-KO) and PRDM16 knock-in (PT-PRDM16-KI) were derived from earlier studies [25]. The ADV-PRDM16 plasmid (5.0 × 10ˆ13 v.g/kg) was administered via a single tail vein injection over three consecutive days [29]. Thereafter, these mice were utilized to establish a sepsis model via cecal ligation and puncture (CLP) [30]. Formononetin/PLGA (formononetin 20 mg/kg) microspheres were injected once through the tail vein 30 min post-CLP. Ultimately, kidney, lung, heart, and liver tissues were harvested at 9 or 18 h post-CLP treatment for subsequent analysis.

### 2.4 Chromatin immunoprecipitation (ChIP) analysis

The ChIP Assay Kit (Catalog Number: P2078, Beyotime Biotechnology, Shanghai, China) was utilized for ChIP analysis. In summary, BUMPT cell lines stably expressing HA-PRDM16-RFP or untreated were exposed to doxycycline (DOX) or NRF2 plasmid with NG or LPS for 24 h. The precipitation of DNA bound to protein was facilitated using HA-tag or NRF2 antibodies, with Mouse IgG as the control. Amplified DNAs were detected via PCR with specific primers: NRF2 (−1392 ∼-1382, WT1): 5′-GTTTGCAGCGTGGACTCATC-3′ (forward) and 5′-ACTTTGCAAGAGGCCAACT G-3′ (reverse); GPX4 (−910 ∼-900, WT1): 5′-TGATGCAAATGGGAATACAGTGG-3′ (forward) and 5′-CCTGAGGCTGGAGTTAGACAC-3′ (reverse); GPX4 (−589 ∼-579, WT2): 5′-CTTCTGGGAAAACTGTCTGGAG-3′ (forward) and 5′-CAGAAGTTGGCCACAGCTG-3′ (reverse); GPX4 (−490 ∼-480, WT3): 5′-TGTTCTCAGACCATCCTAAGGC-3′ (forward) and 5′-TGG CCCTCCAGACAGTTTTC-3′ (reverse).

### 2.5 Luciferase reporter assays

Luciferase vectors for NRF2 (WT1-Luc-NRF2 and WT2-Luc-NRF2), GPX4 (WT1-Luc-GPX4, WT2-Luc-GPX4, WT3-Luc-GPX4, and WT4-Luc-GPX4), mutated NRF2 (MUT1-Luc-NRF2 and MUT2-Luc-NRF2), and GPX4 (MUT1-Luc-GPX4, MUT2-Luc-GPX4, MUT3-Luc-GPX4, and MUT4-Luc-GPX4) were procured from Sangon Biotech. Sequence alignments demonstrated a complementary relationship between NRF2 and GPX4 WT, as well as between PRDM16 and either NRF2 WT or GPX4 WT, while introducing mutations in NRF2 or GPX4 to disrupt this complementarity. The sequences were as follows: NRF2 WT1-5′-CTCATCCATCTCCCTGGGGCAGAG-3′, NRF2 MUT1-5′-CTCATCCATGAGGGACCCCGAGAG-3′, NRF2 WT2-5′’-TTCGGTGC TCCAGAGAACTATAAA-3′, NRF2 MUT2-5′-TTCGGTCGAGGTCTCTTCTATAA A-3′, GPX4 WT1-5′-GGGTGTCCTGTGTCAGGTGTGTCT-3′, GPX4 MUT1-5′-GGGTGAGGACACAGTCGTGTGTCT-3′, GPX4 WT2-5′-CACTGTTCCCTAGTC ACAAGCCTG-3′, GPX4 MUT2-5′-CACTGTAGGGATCAGTGAAGCCTG-3′, GPX4 WT3-5′-AGTCTAAGGCCCAGGAGGATGGAG-3′, GPX4 MUT3-5′-AGTC TAACCGGGTCCTCCATGGAG-3′, GPX4 WT4-5′-TTCAAGTTCACAGGAGAAT CAGAA-3′, GPX4 MUT4-5′-TTCAAGAAGTGTCCTCTATCAGAA-3′. Renilla luciferase (RLuc) was employed as the internal reference for normalization. The pGMLR-TK plasmid was co-transfected with either WT-Luc-NRF2/WT-Luc-GPX4 or MUT-Luc-NRF2/MUT-Luc-GPX4 into the stable HA-PRDM16-RFP-expressing cell line or BUMPT cells, in the presence or absence of DOX or NRF2 plasmid for 24 h. The assay was performed as outlined [31], with reporter activity measured utilizing a SpectraMaxM5 (Molecular Devices) and normalized against RLuc.

### 2.6 Real-time quantitative polymerase chain reaction (RT-qPCR)

Total RNA was isolated from cells and tissues utilizing TRIzol, followed by relative quantification as outlined in prior studies [32]. The primers employed were: Mouse β-actin and homo β-actin, as referenced in a previous study [32]; PRDM16 (Mouse): 5′-CAGCAACCTCCAGCGTCACATC-3′ (forward) and 5′-GCGAAGGTCTTGC CACAGTCAG-3′ (reverse); PRDM16 (Human): 5′-CTTCGGATGGGAGCAA ATACTG-3′ (forward) and 5′-TCCACGCAGAACTTCTCACTG-3′ (reverse); NRF2 (Mouse): 5′-CAACTCGGCGAAGAAAGAAACA-3′ (forward) and 5′-AGGATACTG GGGATTCGTCTG-3′ (reverse); NRF2 (Human): 5′-AACTTTCGGAATTATT GGCAAGC-3′ (forward) and 5′-CGTCTCTGGTCAGATTTGACAGT-3′ (reverse); GPX4 (Mouse): 5′-GCCTGGATAAGTACAGGGGTT-3′ (forward) and 5′-CATGCAG ATCGACTAGCTGAG-3′ (reverse); GPX4 (Human): 5′- GAGGCAAGACCGAAG TAAACTAC-3′ (forward) and 5′- CCGAACTGGTTACACGGGAA-3′ (reverse). Relative quantification was conducted utilizing ΔΔCt values.

### 2.7 Immunoblotting

The Western blotting procedure adhered to the previously established protocol [33]. SDS-PAGE separated identical quantities of cell or tissue protein and subsequently transferred onto PVDF membranes. These membranes were then blocked using 5 % skim milk and incubated at 4 °C overnight with antibodies targeting PRDM16 (1:1000 dilutions), NRF2 (1:1000 dilutions), GPX4 (1:1000 dilutions), COX2 (1:1000 dilutions), NOX1 (1:1000 dilutions), HA (1:2000 dilutions), and β-tubulin (1:2000 dilution). Following this, the membranes were treated with corresponding secondary antibodies (1:5000 dilution, Affinity Biosciences, USA). In this investigation, β-tubulin functioned as the loading reference. Band visualization was accomplished by employing a highly sensitive enhanced chemiluminescent reagent (NCM Biotech, China) on a gel imager (Tanon, China).

### 2.8 Preparation of Formononetin/PLGA microspheres

Initially, 100 mg of PLGA solid was dissolved in 1000 μl of dichloromethane to obtain a PLGA solution at a concentration of 100 mg/ml. Concurrently, formononetin (5.0, 10.0, 15.0, or 25.0 mg) was dissolved in 500 μl of dimethyl sulfoxide (DMSO). The formononetin-DMSO solution was then added to the PLGA solution to form a mixture. This mixture was subsequently introduced into 5 ml of phosphate buffer (10^−3^ M). The resultant mixture was sonicated at an amplitude of 30 % using a probe-type ultrasonic crusher while maintained on ice. The sonicated mixture was gradually dripped into 8 ml of sterile ultrapure water on a magnetic stirrer and stirred for 1–2 h to ensure complete volatilization of the dichloromethane. Finally, the microspheres were collected using a cryogenic centrifuge (5000 rpm), rinsed thrice with phosphate-buffered saline (PBS) solution, dried using a freeze dryer, weighed, and stored at −20 °C for future use. The Scanning Electron Microscope (SEM, HITACHI Regulus 8100, Japan) and Transmission Electron Microscope (TEM, HITACHI HT7800, Japan) were employed to observe and image the microspheres. The diameter of the microspheres was measured using a Nanoparticle Tracking Analyzer (NTA, Particle Metrix, Germany).

### 2.9 Drug loading and encapsulation efficiency

To quantify the drug-loading capacity of PLGA, dried microspheres were initially weighed and then added to 1000 μl of dichloromethane, a solvent in which formononetin is insoluble. Following the complete evaporation of dichloromethane, 500 μl of DMSO was introduced to fully dissolve the formononetin. Subsequently, the supernatant was collected and subjected to centrifugation for analysis via high-performance liquid chromatography (HPLC, Agilent 1260 Infinity Ⅱ, USA). The drug loading (DL) and encapsulation efficiency (EE) were computed utilizing the following equations:

DL (%) = Mf/M1 × 100% (Mf, amount of formononetin in microspheres; M1, microsphere weight.)

EE (%) = Mf/M2 × 100% (Mf, amount of formononetin in microspheres; M2, total amount of formononetin added.)

The actual level of formononetin in formononetin/PLGA microspheres, as determined by DL and EE, was subsequently utilized in animal experiments.

### 2.10 Assessment of glutathione (GSH)/oxidized glutathione (GSSG), Fe^2+^, cell viability, malondialdehyde (MDA), 4-hydroxynonenal (4-HNE), renal function, morphology, immunohistochemistry (IHC) staining, and ROS

GSH and GSSG was quantified utilizing assay kit (Catalog Number: S0053) from Beyotime Biotechnology, Shanghai, China. Ferrous ion content was quantified utilizing assay kit (Catalog Number: BC5415) from Beijing Solarbio Science & Technology Co.,Ltd. (Beijing, China). Cell viability (Catalog Number: G021-1-1), as well as levels of MDA (Catalog Number: A003-1-2), 4-HNE (Catalog Number: H268-1-2), blood urea nitrogen (BUN) (Catalog Number: C013-2-1), and serum creatinine (Catalog Number: C011-2-1), were quantified utilizing assay kits from Nanjing Jiancheng BioEngineering Institute (Jiangsu, China). Histological damage was examined using Hematoxylin and eosin (H&E) staining. Immunohistochemical analysis adhered to a previously established protocol [29,34], employing antibodies against PRDM16 (1:100 dilution), NRF2 (1:100 dilution), and GPX4 (1:100 dilution). The stained samples were subsequently examined utilizing a light microscope (Olympus, Japan) equipped with UV fall illumination. The IHC staining was quantified using the "H-score" method [35]. The TEM was used to observe and image the mitochondria in the proximal tubules of the renal cortex. ROS cells were identified through a ROS detection kit (Abcam, UK) and analyzed utilizing flow cytometry (FCM; Cytek, USA). Dihydroethidium (DHE) staining was used to detect ROS in kidney tissue.

### 2.11 Immunofluorescence

Cells were incubated with specific primary antibodies (PRDM16 at 1:500 dilution, NRF2 at 1:500 dilution, and GPX4 at 1:500 dilution) overnight at 4 °C. Afterwards, specimens were exposed to the corresponding fluorescent secondary antibodies (Abcam, UK) for 1 h at 37 °C in the dark. Finally, DAPI was introduced for 3–5 min, and the cells were visualized using an Eclipse Ti laser scanning confocal microscope (Nikon, Japan).

### 2.12 Statistical analyses

The data were expressed as the mean ± standard deviation (SD). Two-tailed t-tests were used to compare two cohorts, while one-way ANOVA was applied for comparisons among multiple cohorts. Statistical analyses were conducted utilizing SPSS version 25.0 and GraphPad Prism version 9.0 software. A significance level of *p* < 0.05 was considered statistically significant.

## 3 Results

### 3.1 PRDM16 is induced in mouse kidneys and BUMPT cells by CLP and LPS treatment, respectively

The initial investigation was centered on whether PRDM16 expression was elevated during septic AKI, utilizing mouse models of CLP. Blood samples and kidney tissues were procured at 9 and 18 h post-CLP. The serum creatinine and BUN levels were notably elevated at 9 h, reaching their maximum at 18 h (Fig. 1A and B). Histological analysis of H&E-stained renal tissue sections revealed mild to moderate tubular injury at both 9 and 18 h post-CLP (Fig. 1C and D). The mRNA and protein levels of PRDM16 were assessed utilizing RT-qPCR and immunoblotting, showing significant upregulation at 9 h with a peak at 18 h post-CLP (Fig. 1E–G). Immunohistochemical staining (cortex and outer medulla) further corroborated these findings, and also indicating PRDM16's primary localization in the nucleus (Fig. 1H and I, Supplementary Fig. S1). To validate the *in vivo* results, BUMPT cells were subjected to LPS treatment and collected at 6, 18, and 24 h for mRNA and protein expression analysis. PRDM16 expression was induced at 6 h, progressively increased at 12 h, and peaked at 24 h (Fig. 1J-L). Immunofluorescence staining of PRDM16 confirmed the *in vitro* immunoblot results, demonstrating predominant nuclear expression (Fig. 1M and N).

**Fig. 1:**
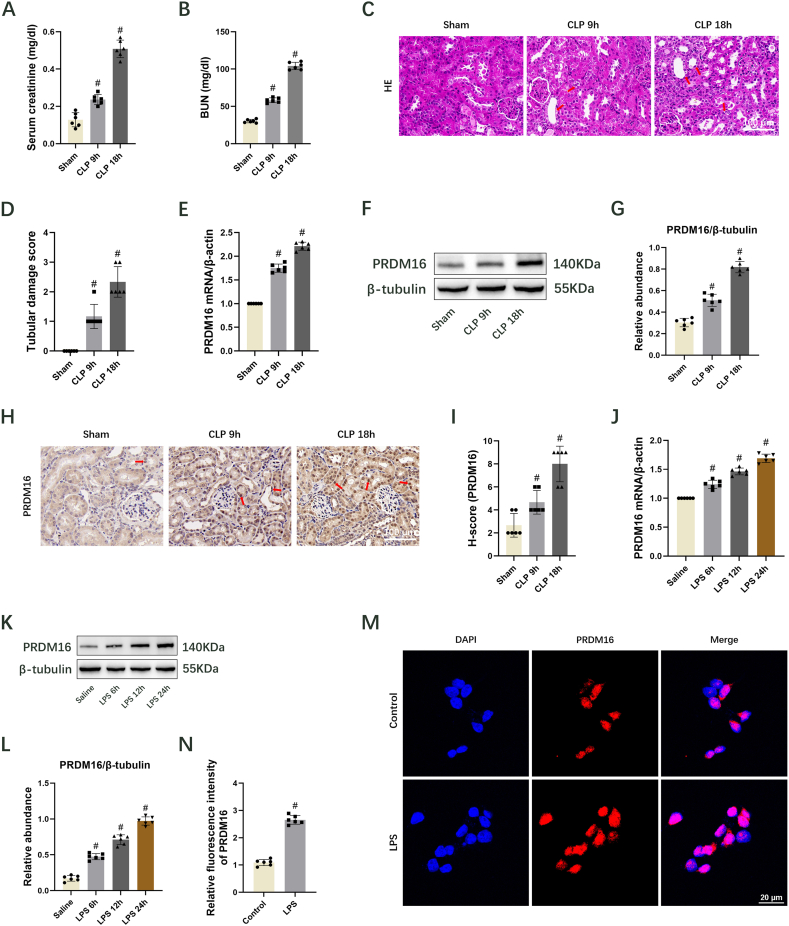
Induction of PRDM16 expression in mouse models and BUMPT cells following sepsis injury. Mice were subjected to 9 or 18 h of CLP. BUMPT cells were exposed to 6, 12, or 24 h of LPS. Mean ± SD (n = 6). (A) Serum creatinine. (B) BUN. (C) H&E staining. The damaged renal tubules were indicated by the arrows. Scale bar: 100 μm. (D) Tubular damage score. (E) RT–qPCR measurement of PRDM16. (F) Immunoblotting showing PRDM16 and β-tubulin. (G) Quantification of immunoblotting. (H) Immunohistochemical staining of PRDM16 in the renal cortex. The nuclei stained positive for PRDM16 were indicated by the arrows. Scale bar: 100 μm. (I) H-score for PRDM16. (J) RT–qPCR measurement of PRDM16. (K) Immunoblotting showing PRDM16 and β-tubulin. (L) Quantification of immunoblotting. (M) The expression and localization of PRDM16 in BUMPT cells were detected by immunofluorescence. Scale bar: 20 μm. (N) Relative fluorescence intensity of PRDM16. ^#^ indicates significance versus Sham, Saline, and Control cohorts, respectively.

### 3.2 PRDM16 suppresses LPS-induced ferroptosis in BUMPT cells

Ferroptosis is pivotal in the context of SA-AKI [36]. It is well known that COX2 [37], NOX1 [38], and GPX4 [39] are key proteins that determine ferroptosis. To investigate the function of PRDM16 in LPS-induced ferroptosis, PRDM16 knockdown was implemented in BUMPT cells, whether or not subjected to a 6 h pretreatment with 1 μM Ferrostatin-1 (Fer-1, Cat.# HY-100579, MedChem Express, China). Results from RT-qPCR and immunoblot analysis indicated a marked reduction in mRNA and protein levels of PRDM16 following transfection with PRDM16 shRNA under both saline (Fig. 2A–F, and G, Supplementary Fig. S5) and LPS conditions (Fig. 2A–F, and G). The suppression of PRDM16 exacerbated the LPS-induced decrease in GSH/GSSG ratio (Fig. 2B), GPX4 (Fig. 2F and G) and cell viability (Supplementary Fig. S2A) while elevating the LPS-induced levels of Fe^2+^ (Fig. 2C), COX2 (Fig. 2F and G), NOX1 (Fig. 2F and G), MDA (Supplementary Fig. 2B), 4-HNE (Supplementary Fig. S2C), and ROS (Fig. 2D and E). However, these effects were reversed by Fer-1 (Fig. 2B–G, Supplementary Figs. S2A–C). Conversely, a BUMPT cell line transfected with HA-PRDM16-RFP was generated, and its expression was induced by DOX treatment. Meantime, with or without subjected to a 24 h pretreatment with 10 μM Erastin (Cat.# HY-15763, MedChem Express, China). As anticipated, PRDM16 overexpression led to elevated mRNA and protein levels of PRDM16 (Fig. 2H, M, and N, Supplementary Fig. S5), enhanced levels of GSH/GSSG ratio (Fig. 2I), GPX4 (Fig. 2M and N) and cell viability (Supplementary Fig. S2D), and reduced production of Fe^2+^ (Fig. 2J), COX2 (Fig. 2M and N), NOX1 (Fig. 2M and N), MDA (Supplementary Fig. S2E), 4-HNE (Supplementary Fig. S2F), and cellular ROS (Fig. 2K and L), while these effects that were reversed by Erastin (Fig. 2I-M, Supplementary Figs. S2D–F). Thus, PRDM16 mitigated LPS-induced ferroptosis by reducing oxidative damage in BUMPT cells.

**Fig. 2:**
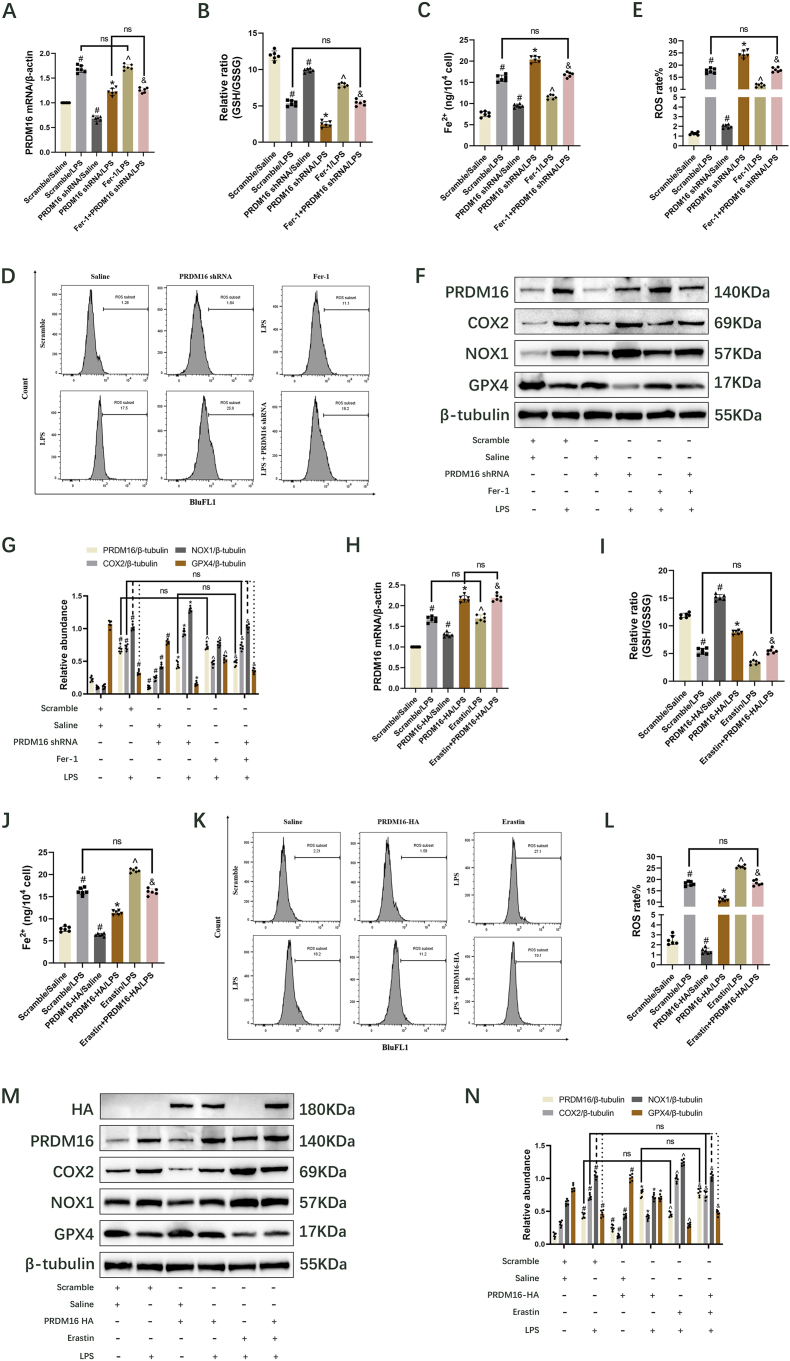
PRDM16 inhibited LPS-induced ferroptosis in BUMPT cells. BUMPT cells or HA-PRDM16-RFP stable BUMPT cell lines were treated with PRDM16 shRNA, DOX, Fer-1, or Erastin respectively, followed by LPS for 24 h. Mean ± SD (n = 6). (A) Expression of PRDM16, shown by RT-qPCR. (B) GSH/GSSG ratio. (C) Levels of Fe^2+^ expression. (D) ROS of cells, shown by FCM. (E) ROS rates. (F) Immunoblotting showing PRDM16, COX2, NOX1, GPX4, and β-tubulin. (G) Quantification of immunoblotting. (H) Expression of PRDM16, shown by RT-qPCR. (I) GSH/GSSG ratio. (J) Levels of Fe^2+^ expression. (K) ROS of cells, shown by FCM. (L) ROS rates. (M) Immunoblotting showing HA, PRDM16, COX2, NOX1, GPX4 and β-tubulin. (N) Quantification of immunoblotting. ^#^, ∗ indicates significance versus Scrambled/Saline and/LPS cohorts, respectively. ^ˆ^ indicates significance versus Scrambled/LPS, PRDM16 shRNA/LPS, and PRDM16-HA/LPS cohorts, respectively. ^&^ indicates significance versus Scrambled/LPS, PRDM16 shRNA/LPS, PRDM16-HA/LPS, Fer-1/LPS, and Erastin/LPS cohorts, respectively.

### 3.3 PRDM16 interacts with the NRF2 promoter to promote its expression

PRDM16, a multifaceted transcriptional regulatory factor, is essential for maintaining the normal physiological function of organisms [40]. NRF2 is implicated in regulating lipid peroxidation and ferroptosis [41]. To elucidate the molecular mechanism by which PRDM16 inhibits ferroptosis, the MoLo Tool (https://molotool.autosome.org/) was utilized to predict PRDM16's binding sites at WT1(−1392–1382) and WT2(−268–258) on the NRF2 promoter (Fig. 3A). Both dual luciferase reporter assay, ChIP assay, and RT-qPCR substantiated that PRDM16 binds to WT1 within the NRF2 promoter region, thereby modulating its expression (Fig. 3B–D). To further investigate the impact of PRDM16 on NRF2, BUMPT cells with either PRDM16 knockdown or overexpression were employed. RT-qPCR and immunoblot analyses demonstrated that PRDM16 knockdown decreased mRNA and protein levels of NRF2 under both saline (Fig. 3E–G and Supplementary Fig. S5) and LPS conditions (Fig. 3E–G). Conversely, PRDM16 overexpression markedly elevated mRNA and protein levels of NRF2 under similar conditions, while these effects were reversed by PRDM16 shRNA (Fig. 3H–J). The findings from immunofluorescence staining of NRF2 corroborated those of RT-qPCR and immunoblot. Furthermore, NRF2 is localized in both the nucleus and cytoplasm, with nuclear translocation observed following LPS treatment (Fig. 3K and L). Thus, PRDM16 positively regulates NRF2 expression.

**Fig. 3:**
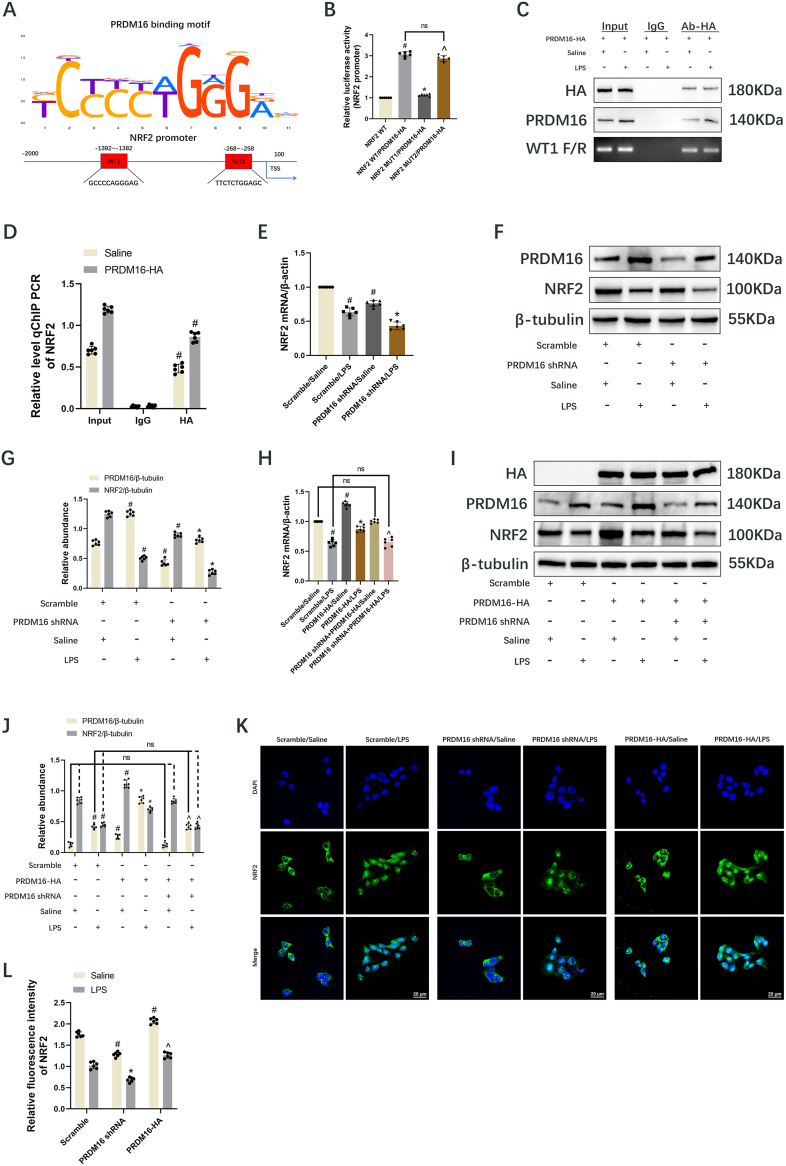
PRDM16 regulates NRF2. HA-PRDM16-RFP stable BUMPT cell lines were treated with DOX followed by saline or LPS for 24 h for ChIP assays using HA antibodies. Mean ± SD (n = 6). (A) Binding site of PRDM16 to the NRF2 promoter. (B) Luciferase activities of co-transfected NRF2 WT1, NRF2 Mut1, NRF2 WT2, or NRF2 Mut2 with DOX. (C) ChIP assays showed that PRDM16 bound to the WT1 (−1 392 ∼-1382 bp) region of the NRF2 gene promoter. (D) Relative level ChIP RT-qPCR of NRF2. (E) Expression of NRF2, shown by RT-qPCR. (F) Immunoblotting showing PRDM16, NRF2, and β-tubulin. (G) Quantification of immunoblotting. (H) RT–qPCR measurement of NRF2. (I) Immunoblotting showing HA, PRDM16, NRF2, and β-tubulin. (J) Quantification of immunoblotting. (K) The expression and localization of NRF2 in BUMPT cells were detected by immunofluorescence. Scale bar: 20 μm. (L) Relative fluorescence intensity of NRF2. ^#^ indicates significance versus NRF2 WT, Scrambled/Saline, Saline/Input, PRDM16-HA/Input, or PRDM16 shRNA + PRDM16-HA/Saline cohorts. ∗ indicates significance versus NRF2 WT1/PRDM16-HA or Scrambled/LPS cohorts. ˆ indicates significance versus NRF2 MUT1/PRDM16-HA, Scrambled/LPS, PRDM16 shRNA/LPS, and PRDM16-HA/LPS cohorts, respectively.

### 3.4 NRF2 interacts with the promotor of GPX4 and activates its expression to inhibit ferroptosis in BUMPT cells during LPS treatment

Numerous studies have demonstrated that NRF2 inhibits ferroptosis through the regulation of GPX4 [[42], [43], [44]]. This study employed JASPAR (https://jaspar.genereg.net/) to predict the relevant binding sites. The prediction revealed that NRF2 binds to the WT1 (−910—900) and WT2 (−589—579) regions of the GPX4 promoter (Fig. 4A). Both dual luciferase reporter assay, ChIP assay, and RT-qPCR verified that NRF2 binds to the WT1 and WT2 regions of the GPX4 promoter (Fig. 4B–D). RT-qPCR and immunoblot analyses indicated that the mRNA and protein levels of GPX4 were markedly decreased following NRF2 knockdown under saline and LPS treatment conditions (Fig. 4E–H and I). Furthermore, NRF2 knockdown exacerbated the LPS-induced reduction in GSH/GSSG ratio (Fig. 4F) and cell viability (Supplementary Fig. S2G), as well as the increase in Fe^2+^ (Fig. 4G), COX2 (Fig. 4H and I), NOX1 (Fig. 4H and I), MDA (Supplementary Fig. 2H), 4-HNE (Supplementary Fig. S2I), and ROS production (Fig. 4O and P). Conversely, overexpression of NRF2 markedly elevated the mRNA and protein levels of GPX4 under saline and LPS treatment conditions (Fig. 4J, M and N). This overexpression also notably reversed the LPS-induced decrease in GSH/GSSG ratio (Fig. 4K) and cell viability (Supplementary Fig. S2J) while suppressing the LPS-induced increase in Fe^2+^ (Fig. 4L), COX2 (Fig. 4M and N), NOX1 (Fig. 4M and N), MDA (Supplementary Fig. S2K), 4-HNE (Supplementary Fig. S2L), and ROS production (Fig. 4O and P). The immunofluorescence staining results for GPX4 were consistent with the RT-qPCR and immunoblot findings (Fig. 4Q and R). Additionally, GPX4 was predominantly localized in the cytoplasm (Fig. 4Q). Therefore, NRF2 positively regulates GPX4 to inhibit ferroptosis during LPS treatment.

**Fig. 4:**
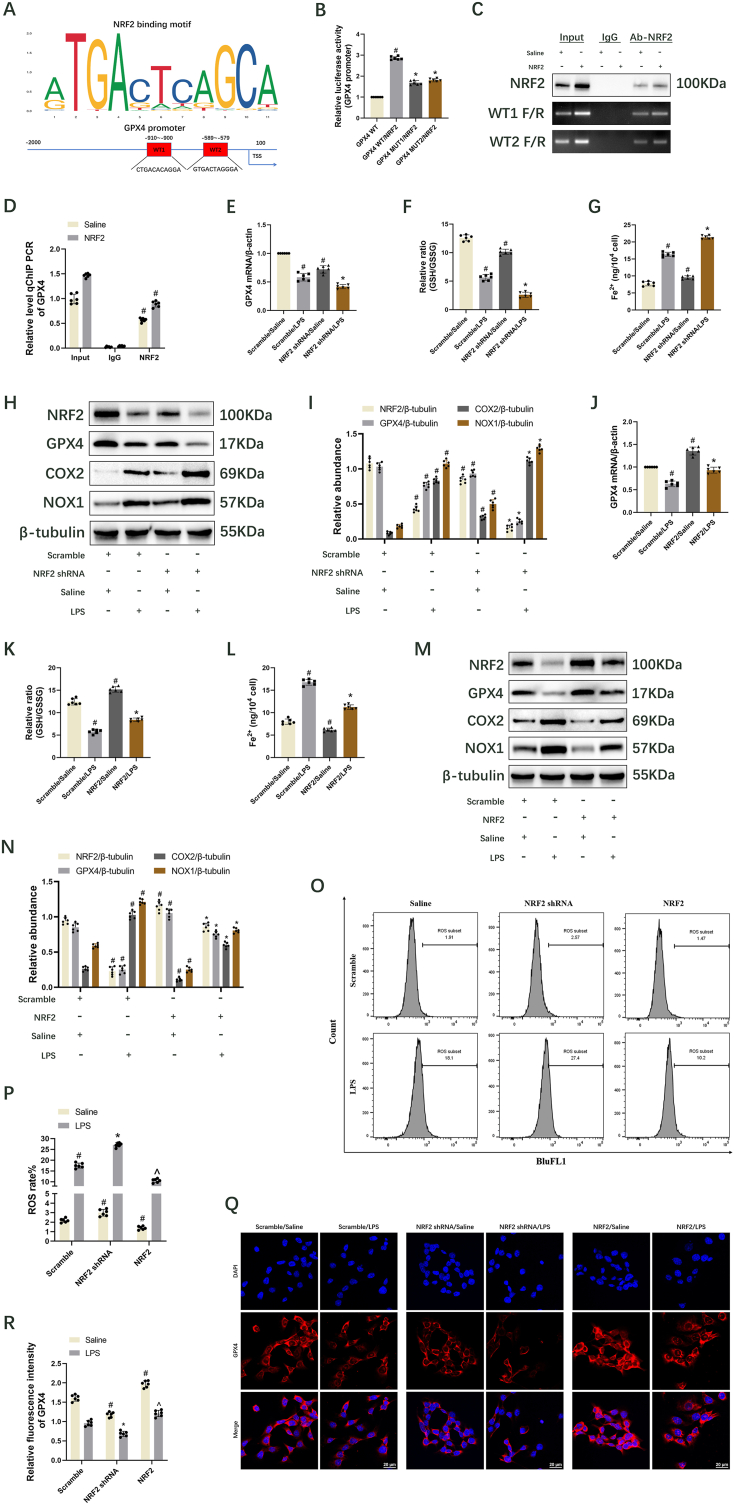
The regulatory role of NRF2 on GPX4 in inhibiting ferroptosis was evaluated. BUMPT cells were subjected to saline or NRF2 plasmid treatment for 24 h, followed by ChIP assays with NRF2 antibodies. Data are presented as Mean ± SD (n = 6). (A) Identification of NRF2 binding site on the GPX4 promoter. (B) Luciferase activity analysis of GPX4 WT1, GPX4 Mut1, GPX4 WT2, or GPX4 Mut2 co-transfected with NRF2 plasmid. (C) ChIP assay results indicate NRF2 binding to WT1 (−910 ∼-900 bp) and WT2 (−589 ∼-579 bp) regions within the GPX4 promoter. (D) Relative level ChIP RT-qPCR of GPX4. (E) GPX4 expression levels measured by RT-qPCR. (F) GSH/GSSG ratio. (G) Levels of Fe^2+^ expression. (H) Immunoblot analysis displaying NRF2, GPX4, COX2, NOX1, and β-tubulin proteins. (I) Immunoblot quantification. (J) GPX4 expression levels measured by RT-qPCR. (K) GSH/GSSG ratio. (L) Levels of Fe^2+^ expression. (M) Immunoblot shows the presence of NRF2, GPX4, COX2, NOX1, and β-tubulin. (N) Quantitative analysis of immunoblot results. (O) ROS levels in cells, as indicated by FCM. (P) ROS rates analysis. (Q) Detection of GPX4 expression and localization in BUMPT cells using immunofluorescence. Scale bar: 20 μm. (R) Relative fluorescence intensity of GPX4. ^#^ denotes significance compared to GPX4 WT, Scrambled/Saline, Saline/Input, or NRF2/Input cohorts. ∗ indicates significance compared to GPX4 WT/NRF2 or Scrambled/LPS cohorts. ˆ denotes significance relative to Scrambled/LPS and NRF2 shRNA/LPS cohorts, respectively.

### 3.5 PRDM16 not only indirectly promotes the GPX4 expression via upregulation of NRF2 but also directly induces the expression of GPX4 to suppress the LPS-induced ferroptosis in BUMPT cells

Rescue studies were conducted to ascertain whether NRF2 mediates PRDM16's effect on ferroptosis. RT-qPCR and immunoblot analyses revealed that PRDM16 knockdown significantly decreased the mRNA and protein levels of PRDM16, NRF2, and GPX4 under both saline and LPS treatment conditions (Fig. 5A–C, H, and I). Overexpression of NRF2 fully restored NRF2 levels (Fig. 5B–H, and I) but only partially restored GPX4 levels (Fig. 5C–H, and I). Furthermore, PRDM16 knockdown intensified the LPS-induced reduction in GSH/GSSG ratio (Fig. 5D) and cell viability (Supplementary Fig. S2M), as well as the increase in Fe^2+^ (Fig. 5E), COX2 (Fig. 5H and I), NOX1 (Fig. 5H and I), MDA (Supplementary Fig. S2N), 4-HNE (Supplementary Fig. S2O), and ROS (Fig. 5F and G), effects that were partially reversed by NRF2 upregulation (Fig. 5D–I, Supplementary Fig. S2M − O). Thus, besides PRDM16 regulating GPX4 expression via NRF2 upregulation, it was hypothesized that PRDM16 might also directly target GPX4. MoLo Tool (https://molotool.autosome.org/) was used to predict PRDM16 binding sites on the GPX4 promoter. The predictions indicated that PRDM16 binds to the WT3(−490–480) and WT4(−399–389) regions of the GPX4 promoter (Fig. 5J). Dual luciferase reporter, ChIP assays, and RT-qPCR confirmed that PRDM16 binds to the WT3 region of the GPX4 promoter to regulate its expression (Fig. 5K-M). Additionally, RT-qPCR and immunoblot results showed that PRDM16 upregulation markedly elevated the mRNA and protein levels of PRDM16 and NRF2 under both saline and LPS treatment conditions (Fig. 5N and O, Q, and R). However, PRDM16 overexpression reversed the LPS-induced reduction in GPX4 (Fig. 5P-R) and increase in COX2 and NOX1 (Fig. 5Q and R), though this effect was partially diminished by NRF2 shRNA (Fig. 5P-R). In conclusion, PRDM16 suppresses LPS-induced ferroptosis by targeting NRF2 to regulate GPX4 and by directly targeting GPX4.

**Fig. 5:**
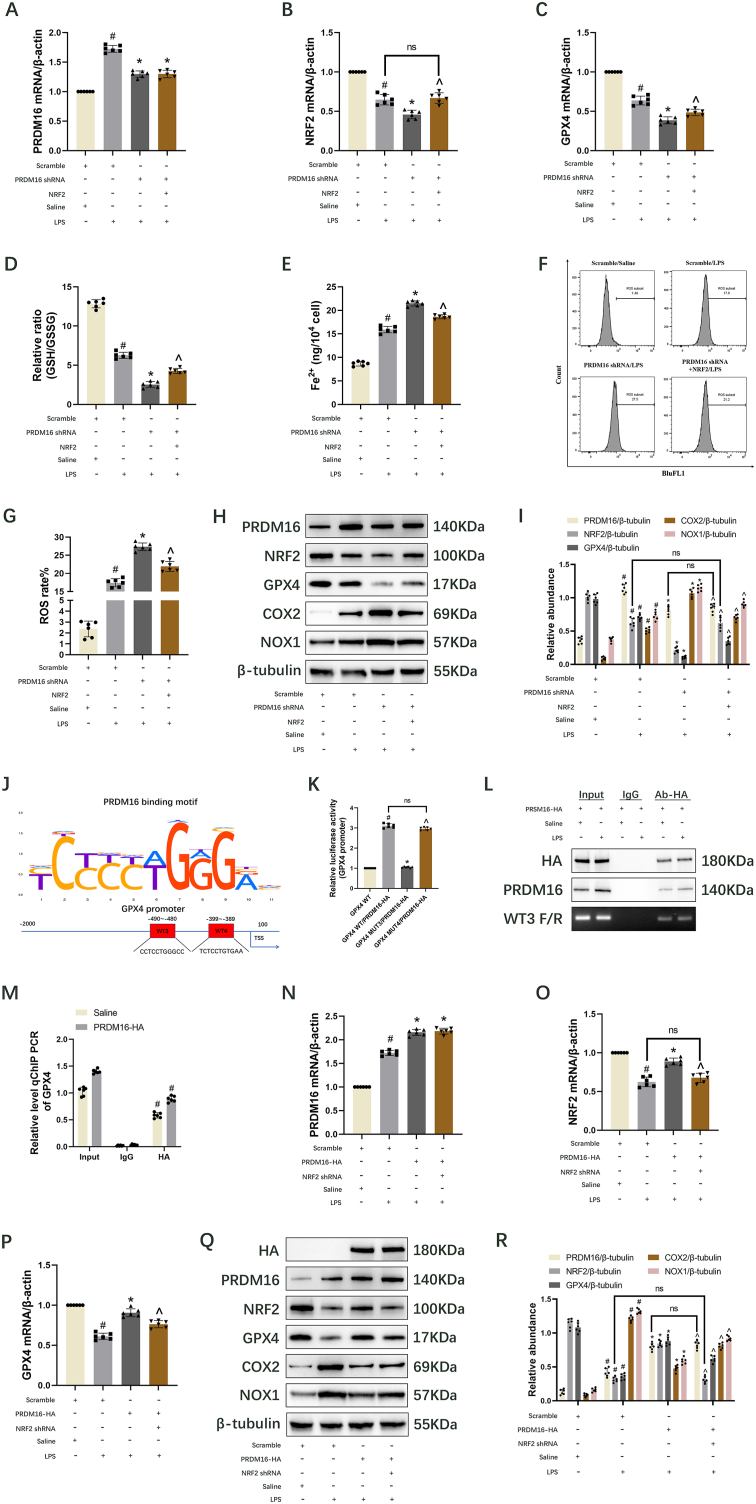
PRDM16 modulates the NRF2/GPX4 or GPX4 axis to facilitate LPS-induced ferroptosis in BUMPT cells. After transfection with PRDM16 shRNA or DOX, in the presence or absence of NRF2 plasmid or shRNA, cells underwent a 24 h LPS exposure. Data are presented as Mean ± SD (n = 6). (A–C) RT-qPCR quantification of PRDM16, NRF2, and GPX4. (D) GSH/GSSG ratio. (E) Levels of Fe^2+^ expression. (F) ROS levels in cells, determined by FCM. (G) ROS rate analysis. (H) Immunoblot analysis displaying PRDM16, NRF2, GPX4, COX2, NOX1, and β-tubulin. (I) Immunoblot quantification. (J) Identification of PRDM16 binding site on the GPX4 promoter. (K) Analysis of luciferase activities in cells co-transfected with GPX4 WT3, GPX4 Mut3, GPX4 WT4, or GPX4 Mut4 with DOX. (L) ChIP assays confirmed PRDM16 binding to the WT3 (−490 ∼-480 bp) region of the GPX4 promoter. (M) Relative level ChIP RT-qPCR of GPX4. (N–P) RT-qPCR quantification of PRDM16, NRF2, and GPX4. (Q) Immunoblot analysis of HA, PRDM16, NRF2, GPX4, COX2, NOX1, and β-tubulin. (R) Quantification of immunoblot results. ^#^ denotes significance compared to Scrambled/Saline, GPX4 WT, Saline/Input, or PRDM16-HA/Input cohorts. ∗ indicates significance compared to GPX4 WT/PRDM16-HA or Scrambled/LPS cohorts. ˆ denotes significance relative to Scrambled/LPS, PRDM16 shRNA/LPS, GPX4 WT/PRDM16-HA, GPX4 MUT3/PRDM16-HA, or PRDM16-HA/LPS cohorts.

### 3.6 The knockout of PRDM16 in the proximal tubular promotes ferroptosis to aggravate septic AKI via inhibition of the NFR2/GPX4 axis

**Fig. 6:**
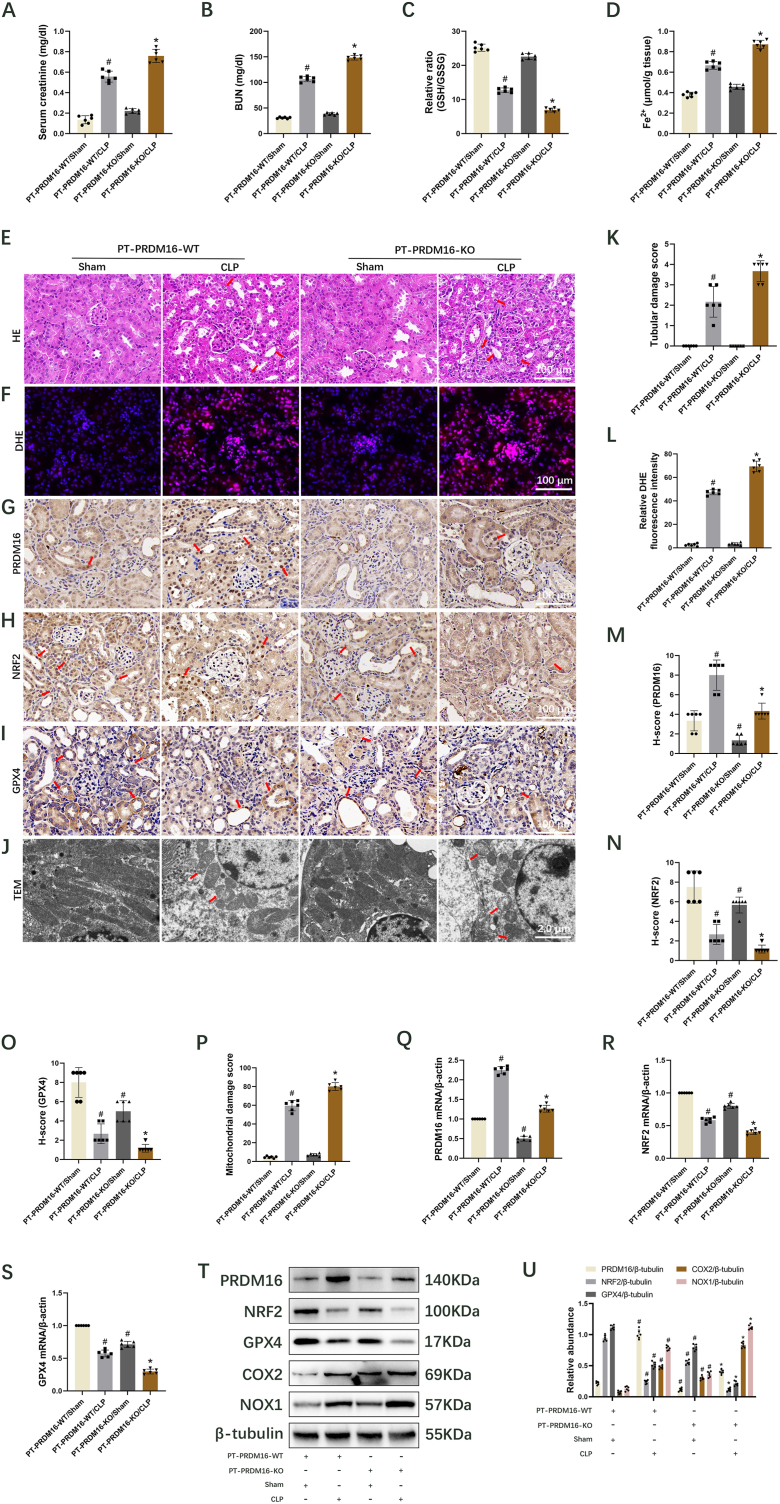
PT-PRDM16-KO exacerbates sepsis-induced AKI by promoting ferroptosis through the NRF2/GPX4 axis. Mouse models of CLP were established, and blood samples and kidney tissues were collected 18 h post-CLP. Data are presented as Mean ± SD (n = 6). (A) Serum creatinine. (B) BUN levels. (C) GSH/GSSG ratio. (D) Levels of Fe^2+^ expression. (E) H&E staining (Scale bar: 100 μm). The damaged renal tubules were indicated by the arrows. (F) DHE staining (Scale bar: 100 μm). (G) Immunohistochemical staining of PRDM16 (Scale bar: 100 μm). The nuclei stained positive for PRDM16 were indicated by the arrows. (H) Immunohistochemical staining of NRF2 (Scale bar: 100 μm). The nuclei stained positive for NRF2 were indicated by the arrows. (I) Immunohistochemical staining of GPX4 (Scale bar: 100 μm). The renal tubules stained positive for GPX4 were indicated by the arrows. (J) TEM images of the renal cortex. (Scale bar: 2.0 μm). The damaged mitochondria were indicated by the arrows. (K) Tubular damage score. (L) Relative DHE fluorescence intensity. (M) H-score for PRDM16. (N) H-score for NRF2. (O) H-score for GPX4. (P) Mitochondrial damage score. (Q–S) RT–qPCR quantification of PRDM16, NRF2, and GPX4. (T) Immunoblotting of PRDM16, NRF2, GPX4, COX2, NOX1, and β-tubulin. (U) Quantification of immunoblotting results. ^#^, ∗ indicate significance versus PT-PRDM16-WT/Sham and/CLP cohorts, respectively.

To examine the function of PRDM16 in renal proximal tubules, PT-PRDM16-WT and KO mice were administered CLP treatment. Blood samples and kidney tissues were obtained 18 h post-CLP. Renal function analysis indicated that PT-PRDM16-KO mice exhibited an enhanced CLP-induced increase in serum creatinine and BUN levels (Fig. 6A and B). Concurrently, PT-PRDM16-KO mice demonstrated a significant CLP-induced reduction in GSH/GSSG ratio (Fig. 6C) and upregulation of Fe^2+^ (Fig. 6D), MDA (Supplementary Fig. S2P), 4-HNE (Supplementary Fig. S2Q), COX2 (Fig. 6T and U), NOX1 (Fig. 6T and U), tubular injury (Fig. 6E and K), and ROS (Fig. 6F and L). Immunohistochemistry staining revealed that PT-PRDM16-KO further decreased PRDM16, NRF2, and GPX4 levels following sham and CLP (Fig. 6G–I and M − O), which was corroborated by RT-qPCR (Fig. 6Q–S) and immunoblot (Fig. 6T and U, Supplementary Fig. S5) results. Additionally, TEM analysis of kidney tissues showed that PT-PRDM16-KO markedly intensified CLP-induced mitochondrial damage (Fig. 6J and P). Collectively, these findings suggest that PT-PRDM16-KO promotes ferroptosis, thereby worsening sepsis-induced AKI via the NRF2/GPX4 axis.

### 3.7 The knock-in of PRDM16 in the proximal tubular inhibits the ferroptosis to alleviate sepsis-induced mice AKI via upregulation of NRF2/GPX4 axis

**Fig. 7:**
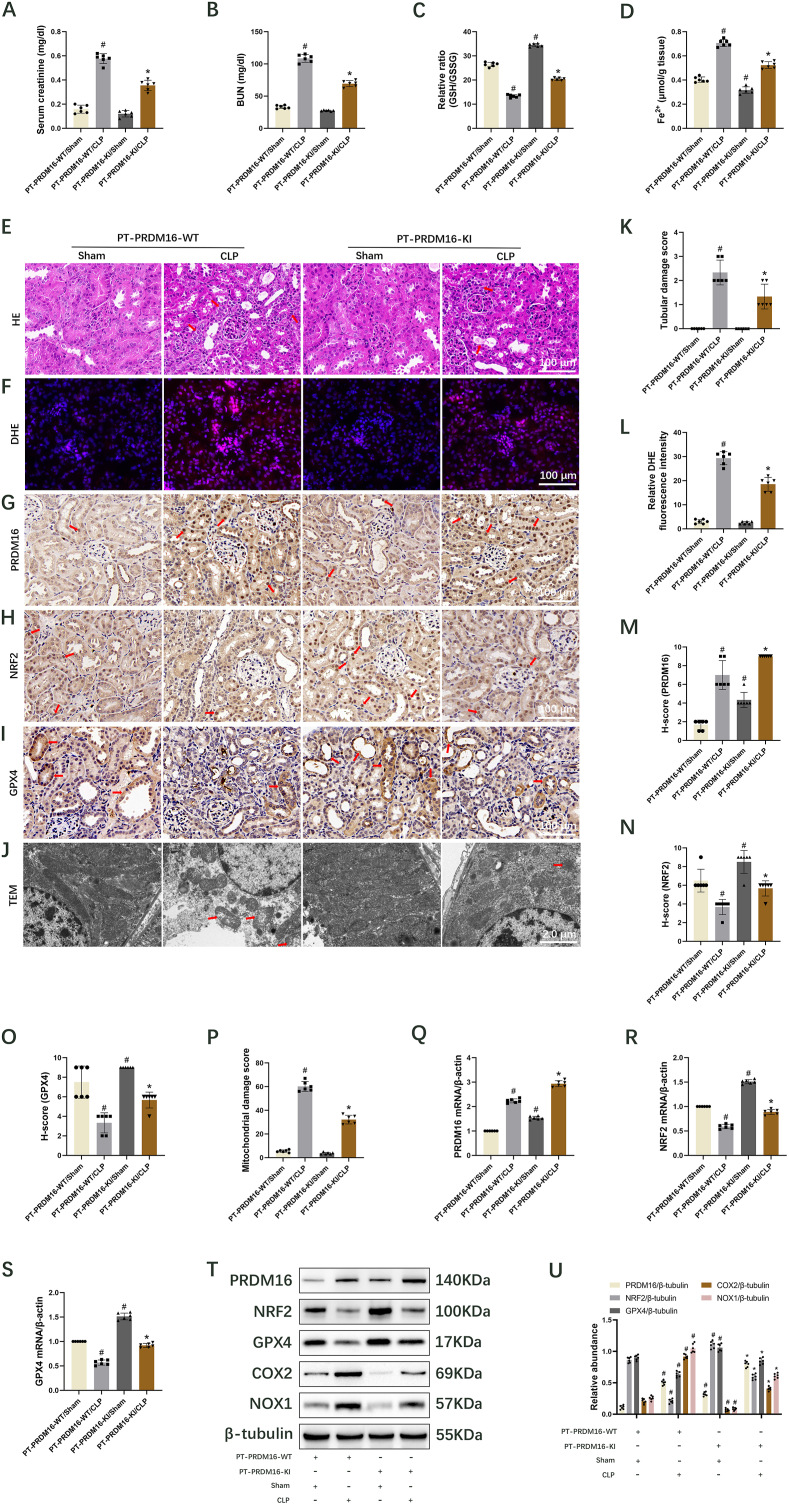
PT-PRDM16-KI inhibits ferroptosis through the NRF2/GPX4 pathway to alleviate sepsis-induced AKI. Mouse models of CLP were created, and blood samples and kidney tissues were collected 18 h post-CLP. Data are presented as Mean ± SD (n = 6). (A) Serum creatinine. (B) BUN levels. (C) GSH/GSSG ratio. (D) Levels of Fe^2+^ expression. (E) H&E staining (Scale bar: 100 μm). The damaged renal tubules were indicated by the arrows. (F) DHE staining (Scale bar: 100 μm). (G) Immunohistochemical staining of PRDM16 (Scale bar: 100 μm). The nuclei stained positive for PRDM16 were indicated by the arrows. (H) Immunohistochemical staining of NRF2 (Scale bar: 100 μm). The nuclei stained positive for NRF2 were indicated by the arrows. (I) Immunohistochemical staining of GPX4 (Scale bar: 100 μm). The renal tubules stained positive for GPX4 were indicated by the arrows. (J) TEM images of the renal cortex. (Scale bar: 2.0 μm). The damaged mitochondria were indicated by the arrows. (K) Tubular damage score. (L) Relative DHE fluorescence intensity. (M) H-score for PRDM16. (N) H-score for NRF2. (O) H-score for GPX4. (P) Mitochondrial damage score. (Q–S) RT-qPCR quantification of PRDM16, NRF2, and GPX4. (T) Immunoblotting showing PRDM16, NRF2, GPX4, COX2, NOX1, and β-tubulin. (U) Quantification of immunoblotting results. ^#^, ∗ indicate significance compared to PT-PRDM16-KI/Sham and/CLP cohorts, respectively.

To further elucidate the role of PRDM16, PT-PRDM16-KI and WT mice underwent CLP procedure. Blood samples and kidney tissues were obtained 18 h post-CLP. PT-PRDM16-KI mice demonstrated suppression of the CLP-induced increases in serum creatinine and BUN levels (Fig. 7A and B). Additionally, PT-PRDM16-KI significantly reversed the CLP-induced decrease in GSH/GSSG ratio (Fig. 7C) while suppressing the CLP-induced increase in Fe^2+^ (Fig. 7D), MDA (Supplementary Fig. S2R), 4-HNE (Supplementary Fig. S2S), COX2 (Fig. 7T and U), NOX1 (Fig. 7T and U), renal tubular injury (Fig. 7E and K), and ROS (Fig. 7F and L). Immunohistochemical staining revealed that PT-PRDM16-KI elevated the levels of PRDM16, NRF2, and GPX4 following sham and CLP (Fig. 7G–I and M − O), a finding that was corroborated by RT-qPCR (Fig. 7Q–S) and immunoblot (Fig. 7T and U, Supplementary Fig. S5) results. Finally, TEM analysis of kidney tissues indicated that PT-PRDM16-KI noticeably alleviated CLP-induced mitochondrial damage (Fig. 7J and P). These data suggest that PT-PRDM16-KI inhibits ferroptosis to attenuate septic AKI via upregulation of the NRF2/GPX4 axis.

### 3.8 ADV-PRDM16 plasmid suppresses ferroptosis to attenuate multiple organ injury in septic mice by upregulation of NRF2/GPX4 axis

**Fig. 8:**
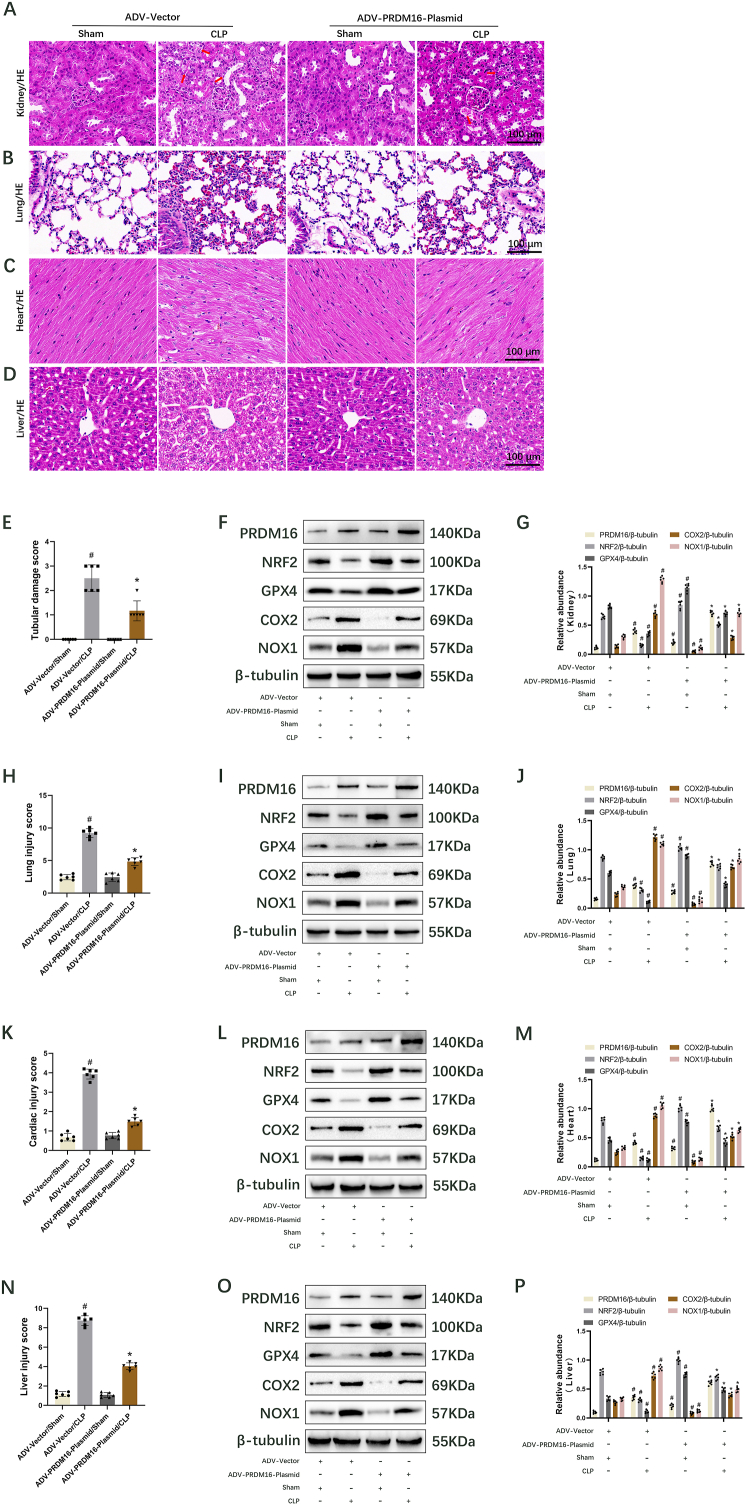
ADV-PRDM16 plasmid alleviates sepsis-induced multi-organ damage in mice through the NRF2/GPX4 axis. The ADV-PRDM16 plasmid was administered to mice 3 days before modeling, with control mice receiving ADV-Vector, followed by CLP for 18 h. Data are presented as Mean ± SD (n = 6). (A–D) H&E staining. The damaged renal tubules were indicated by the arrows. Scale bar: 100 μm. (E) Tubular damage score. (F) Immunoblotting shows PRDM16, NRF2, GPX4, COX2, NOX1, and β-tubulin in the kidney. (G) Quantification of immunoblotting results. (H) Lung injury score. (I) Immunoblotting shows PRDM16, NRF2, GPX4, COX2, NOX1, and β-tubulin in the lung. (J) Quantification of immunoblotting results. (K) Cardiac injury score. (L) Immunoblotting shows PRDM16, NRF2, GPX4, COX2, NOX1, and β-tubulin in the heart. (M) Quantification of immunoblotting results. (N) Liver injury score. (O) Immunoblotting shows PRDM16, NRF2, GPX4, COX2, NOX1, and β-tubulin in the liver. (P) Quantification of immunoblotting results. ^#^, ∗ indicate significance compared to ADV-Vector/Sham and/CLP cohorts, respectively.

PRDM16 is ubiquitously expressed across tissues and organs [45]. To investigate the impact of PRDM16 on various organs, including the kidneys, ADV-PRDM16 plasmids were administered via the tail vein three days before the CLP model, with control mice receiving saline followed by CLP for 18 h. H&E staining of tissue sections revealed that PRDM16 overexpression significantly ameliorated CLP-induced damage in renal tubules (Fig. 8A and E), alveoli (Fig. 8B and H), myocardium (Fig. 8C and K), and liver (Fig. 8D and N). Immunoblot analysis demonstrated that PRDM16 overexpression markedly elevated the protein levels of PRDM16, NRF2, and GPX4, but significantly decreased the protein levels of COX2 and NOX1 in the kidney (Fig. 8F and G, Supplementary Fig. S5), lung (Fig. 8I and J), heart (Fig. 8L and M), and liver (Fig. 8O and P) tissues following sham and CLP. In summary, PRDM16 overexpression mitigates sepsis-induced multi-organ injury via upregulation of the NRF2/GPX4 axis.

### 3.9 Formononetin/PLGA inhibits the ferroptosis to alleviate multiple organ injury in septic mice by upregulation of NRF2/GPX4

**Fig. 9:**
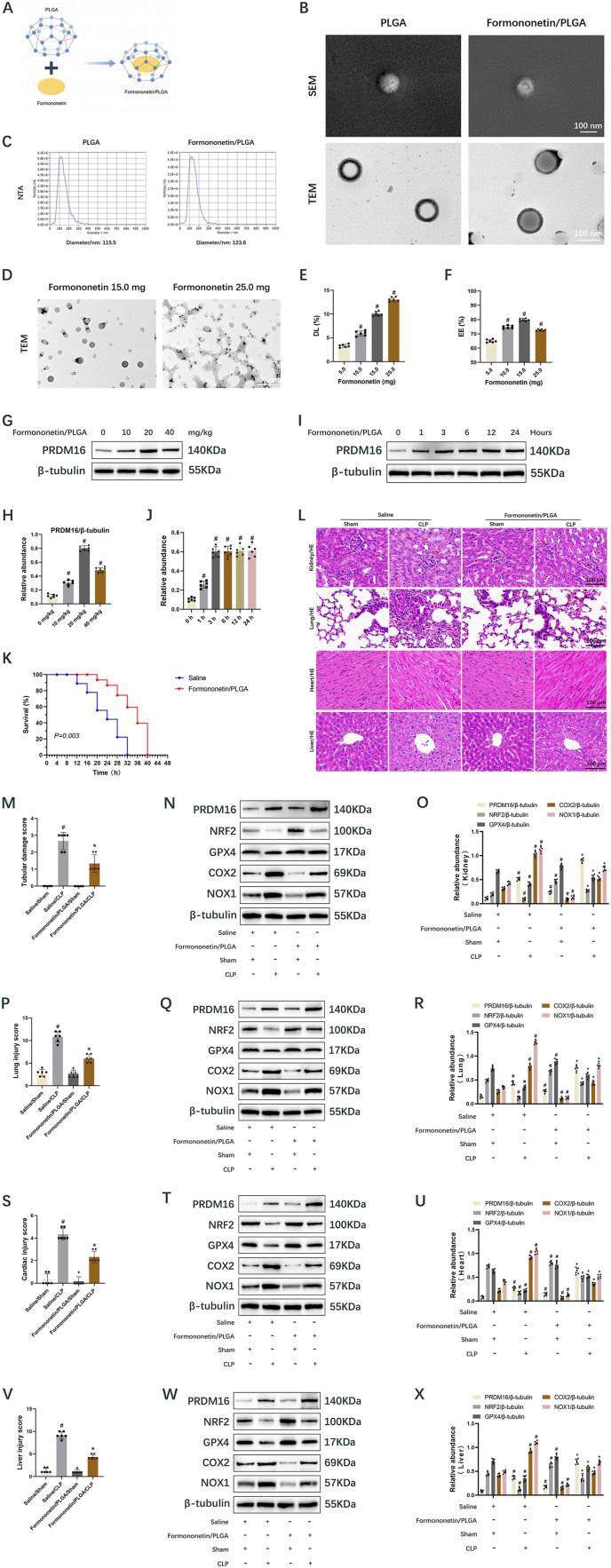
Formononetin/PLGA attenuates sepsis-induced multi-organ damage in mice through the NRF2/GPX4 axis. Initially, PLGA nanoparticles were utilized to formulate Formononetin/PLGA microspheres. Formononetin/PLGA were then administered to the mice via the tail vein 30 min post-CLP, while control mice received saline followed by CLP for 18 h. Data are presented as Mean ± SD (n = 6). (A) Schematic depiction of the Formononetin/PLGA preparation. (B) SEM and TEM images of PLGA and Formononetin/PLGA. Scale bar: 100 nm. (C) NTA results for PLGA and Formononetin/PLGA. (D) TEM image of Formononetin/PLGA. Scale bar: 500 nm. (E) Drug loading capacity of PLGA. (F) Encapsulation efficiency of PLGA. (G) Immunoblot analysis showing PRDM16 and β-tubulin. (H) Quantification of immunoblot results. (I) Immunoblot analysis showing PRDM16 and β-tubulin. (J) Quantification of immunoblot results. (K) Survival rate of mice. (L) H&E staining. The damaged renal tubules were indicated by the arrows. Scale bar: 100 μm. (M) Tubular damage score. (N) Immunoblot analysis showing PRDM16, NRF2, GPX4, COX2, NOX1, and β-tubulin in the kidney. (O) Quantification of immunoblot results. (P) Lung injury score. (Q) Immunoblot analysis showing PRDM16, NRF2, GPX4, COX2, NOX1, and β-tubulin in the lung. (R) Quantification of immunoblot results. (S) Cardiac injury score. (T) Immunoblot analysis showing PRDM16, NRF2, GPX4, COX2, NOX1, and β-tubulin in the heart. (U) Quantification of immunoblot results. (V) Liver injury score. (W) Immunoblot analysis showing PRDM16, NRF2, GPX4, COX2, NOX1, and β-tubulin in the liver. (X) Quantification of immunoblot results. ^#^ indicates significance versus 5.0 mg, 0 mg/kg, 0 h, and Saline/Sham cohorts, respectively. ∗ indicates significance versus Saline/CLP.

Our recent study found that formononetin, a phytoestrogen extracted from red clover leaves, binds and activates the expression of PRDM16 in DKD [25]. Additionally, formononetin has been reported to reduce organ injury in sepsis via gavage administration [46,47]. However, the bioavailability of formononetin is limited and has been used only for prevention, not for treatment. As an advanced nanocarrier, PLGA nanoparticles were employed to prepare Formononetin/PLGA microspheres using the classical “water/oil” method for encapsulating hydrophobic formononetin (Fig. 9A). SEM and TEM indicated successful encapsulation of formononetin by PLGA (Fig. 9B). The average diameter of PLGA microspheres was ∼115.5 nm, whereas the average diameter of Formononetin/PLGA microspheres was ∼123.6 nm, as measured by NTA (Fig. 9C). Efforts to enhance the DL and EE of Formononetin/PLGA microspheres by increasing the drug dosage were made; however, at dosages of 5.0 and 10.0 mg, the DL and EE remained insufficiently low (Fig. 9E and F). Compared to the Formononetin (15 mg dose)/PLGA microspheres, Formononetin (25 mg dose)/PLGA microspheres exhibited lower EE and incomplete morphology despite higher DL (Fig. 9D–F). Thus, the optimal formononetin dose for preparing Formononetin/PLGA microspheres was determined to be 15.0 mg. According to DL and EE, ∼12.0 mg of formononetin was contained in the Formononetin/PLGA microspheres. Subsequently, Formononetin/PLGA microspheres were injected once through the tail vein of mice at doses of 0, 10, 20, and 40 mg/kg of formononetin for 24 h, respectively. Immunoblot results revealed that PRDM16 expression was induced at 10 mg/kg, peaked at 20 mg/kg, and gradually decreased at 40 mg/kg (Fig. 9G and H). Therefore, Formononetin/PLGA microspheres at a dose of 20 mg/kg formononetin were injected into mice via the tail vein. Immunoblot results showed induction of PRDM16 expression at 1 h, peaking at 3 h, and maintaining steady expression at 6, 12, and 24 h (Fig. 9I and J). Formononetin/PLGA microspheres (formononetin 20 mg/kg) were administered to mice via the tail vein 30 min after CLP, while the control mice received saline, followed by CLP for 18 h. Formononetin/PLGA significantly improved the survival rate of mice following CLP (Fig. 9K). H&E staining of tissue sections demonstrated that Formononetin/PLGA significantly ameliorated CLP-induced injuries in renal tubules (Fig. 9L and M), alveoli (Fig. 9L and P), myocardium (Fig. 9L and Q), and liver (Fig. 9L and V). Immunoblot results showed that Formononetin/PLGA markedly increased the protein levels of PRDM16, NRF2, and GPX4, while significantly decreased the protein levels of COX2 and NOX1 in kidney (Fig. 9N and O, Supplementary Fig. S5), lung (Fig. 9Q and R), heart (Fig. 9T and U), and liver (Fig. 9W and X) tissues after sham and CLP. These findings suggest that formononetin inhibits ferroptosis to treat sepsis-induced multiple organ injury by targeting the PRDM16/NRF2/GPX4 axis.

## 4 Discussion

Our recent investigation demonstrated that PRDM16 suppresses the progression of DKD [25]. The current research presents novel findings indicating that PRDM16 suppressed ferroptosis to ameliorate sepsis-induced multiple organ injury, including AKI. Mechanistically, PRDM16 binds to the promoter regions of NRF2 or GPX4 to transactivate their expression, ultimately promoting GPX4 expression to suppress ferroptosis. Notably, the overexpression of PRDM16 or PLGA-encapsulated formononetin suppressed ferroptosis, thereby attenuating multi-organ injury, including AKI, in septic mice. Collectively, these findings reveal that PRDM16 is a novel suppressor of ferroptosis and that targeting PRDM16 using PLGA-encapsulated formononetin represents a new therapeutic strategy for sepsis-induced multiple organ injury, including AKI.

PRDM16 was first identified in acute myeloid leukemia [48]. Recent research has demonstrated that PRDM16 is involved not only in fat metabolism but also in diabetes, cardiomyopathy, cancer, aneurysm, and renal fibrosis [21,25,[49], [50], [51]]. In our previous study, NF-*κ*B was found to mediate the upregulation of PRDM16, thereby inhibiting renal fibrosis through the transient receptor potential ankyrin 1 (TRPA1)/mitogen-activated protein kinase (MAPK)/transforming growth factor-β1 (TGF-β1) axis [25]. The pivotal role of NF-*κ*B in the pathogenesis of sepsis is widely acknowledged [52]. Accumulating evidence suggests a strong association between sepsis and fatty acid oxidation [53], with PRDM16 emerging as a pivotal regulator of fatty acid oxidation [21]. PRDM16 could promote fat oxidation and thermogenesis by up-regulating uncoupling protein 1 (UCP1) [22]. Study has reported that PRDM16 targets and regulates the transcriptional activity of peroxisome proliferator activated receptor alpha (PPAR-α) and peroxisome proliferator-activated receptor-gamma coactivator-1 alpha (PGC-1α), thereby protecting cardiomyocytes from mitochondrial damage in type 2 diabetic cardiomyopathy [24]. Interestingly, UCP1, PPAR-α, and PGC-1α are closely associated with sepsis-related mitochondrial damage [[54], [55], [56]]. The collective evidence suggests a potential association between PRDM16 and sepsis. The current investigation indicates that PRDM16 expression is elevated in mouse kidneys, BUMPT, and HK-2 cells following sepsis injury. We subsequently demonstrated, for the first time, that PRDM16 exerts an inhibitory effect on ferroptosis to confer protection against sepsis-associated AKI by targeting the NRF2/GPX4 axis. Furthermore, the overexpression of PRDM16 attenuated ferroptosis in CLP-induced multiple organ injury, including AKI.

Ferroptosis, a newly characterized form of cell death, is driven by iron dependency and lipid peroxidation [11]. GPX4 impedes ferroptosis through the elimination of various oxidative substrates [57]. As a transcription factor, NRF2 transcribes and regulates almost all ferroptosis-related genes, including GPX4 [58]. Additionally, numerous studies have demonstrated that activation of the NRF2/GPX4 axis inhibits ferroptosis in hepatocytes, cardiomyocytes, nerve cells, and alveolar epithelial cells [[59], [60], [61], [62]]. In alignment with these findings, we confirmed that NRF2 enhances GPX4 expression, thereby inhibiting ferroptosis in BUMPT cells post-LPS exposure. Prior studies have indicated that NRF2 transcription is influenced by multiple transcription factors, such as aryl hydrocarbon receptor (AHR), nuclear factor (NF)-κB, Kirsten rat sarcoma viral oncogene homolog (KRAS), B-Raf, Myc, phosphoinositide 3-kinase (PI3K)/threonine protein kinase (AKT), and Notch [[63], [64], [65], [66], [67]]. In this investigation, we discovered that PRDM16 positively regulates NRF2, a finding supported by transcription binding site predictions, luciferase reporter assays, and ChIP analysis. Consequently, it was revealed that PRDM16 inhibits ferroptosis through the upregulation of the NRF2/GPX4 axis. Intriguingly, rescue studies identified GPX4 as a potential direct downstream target of PRDM16, which was corroborated by transcription binding site predictions, luciferase reporter assays, and ChIP analysis. Overall, our findings demonstrated that PRDM16 suppresses ferroptosis via the NRF2/GPX4 axis or directly through GPX4, as confirmed by PT-PRDM16-KO, PT-PRDM16-KI, and ADV-PRDM16 plasmid experiments in sepsis mice induced by CLP.

Recent investigations have indicated that formononetin mitigates sepsis-induced organ damage by activating the hnRNPUL2/NLRP3 and PI3K/AKT pathways [46,47]. Our previous study reported that formononetin directly binds to and upregulates PRDM16 expression, subsequently inhibiting renal fibrosis in diabetic mice [25]. Nevertheless, the clinical use of formononetin is restricted due to its poor aqueous solubility and low bioavailability [68,69]. Consequently, PLGA encapsulation is employed to improve its solubility and bioavailability. In our research, it was demonstrated for the first time that PLGA-encapsulated formononetin upregulates PRDM16, which activates the NRF2/GPX4 pathway, thereby inhibiting ferroptosis and reducing sepsis-induced multi-organ injury, including AKI. However, the protective effect of Formononetin/PLGA failed to function in the CLP model of PT-PRDM16-KO mice (Supplementary Fig. S4).

Despite the encouraging results of our study, several limitations remain. First, our findings have not been validated in patients with sepsis. Secondly, the preparation process of formononetin microspheres needs to be further optimized to achieve the best parameters. Finally, sepsis-related organ damage is an extremely complex process that needs us to constantly explore and discover.

**Fig. 10:**
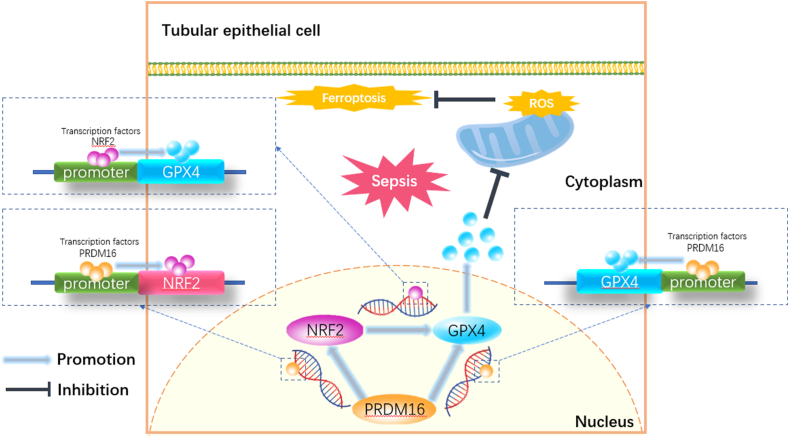
Upon sepsis-induced AKI, PRDM16 targets the NRF2/GPX4 axis, resulting in upregulation of GPX4 and inhibition of ferroptosis, thereby mitigating damage to renal tubular epithelial cells.

In conclusion, our study presents the initial evidence that PRDM16 suppresses LPS or CLP-induced ferroptosis both *in vitro* and *in vivo*. Mechanistically, PRDM16 binds directly to the promoter regions of NRF2 and GPX4, enhancing their expression, which in turn induces GPX4 expression (Fig. 10). Finally, it was found that ADV-PRDM16 and PLGA-encapsulated formononetin represent promising therapeutic approaches for CLP-induced multi-organ injury, including AKI.

## CRediT authorship contribution statement

**Qiang Zheng:** Investigation, Software, Writing – original draft, Conceptualization, Formal analysis, Visualization, Data curation. **Jihong Xing:** Conceptualization, Project administration, Resources, Writing – review & editing. **Xiaozhou Li:** Data curation, Software. **Xianming Tang:** Investigation, Validation. **Dongshan Zhang:** Methodology, Supervision, Writing – review & editing, Project administration, Validation, Visualization.

## Ethics approval and consent to participate

All animal testing procedures were carried out in accordance with the approved ethical guidelines set by the Animal Care Ethics Committee of Second Xiangya Hospital (NO. 2018065).

## Data availability

The corresponding author can provide the datasets used and/or analyzed in this study upon receiving a reasonable request.

## Declaration of competing interest

The authors declare that they have no known competing financial interests or personal relationships that could have appeared to influence the work reported in this paper.

## Figures and Tables

**figs1:**
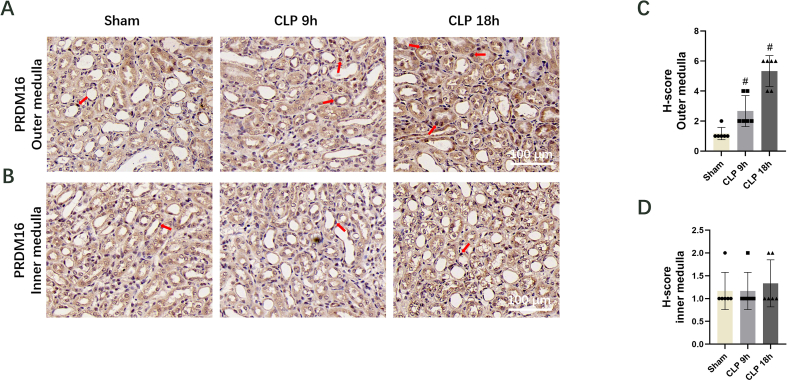


**figs2:**
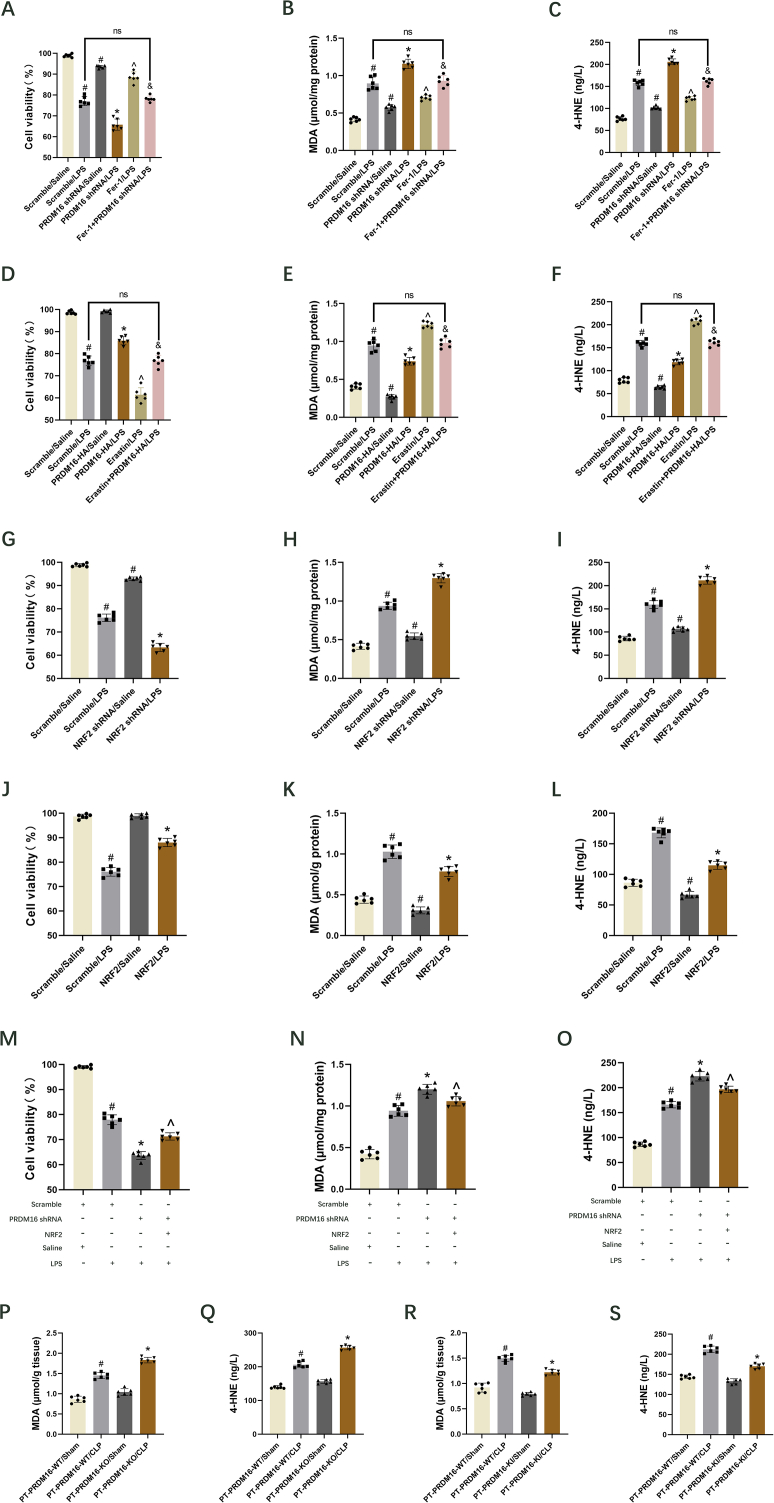


**figs3:**
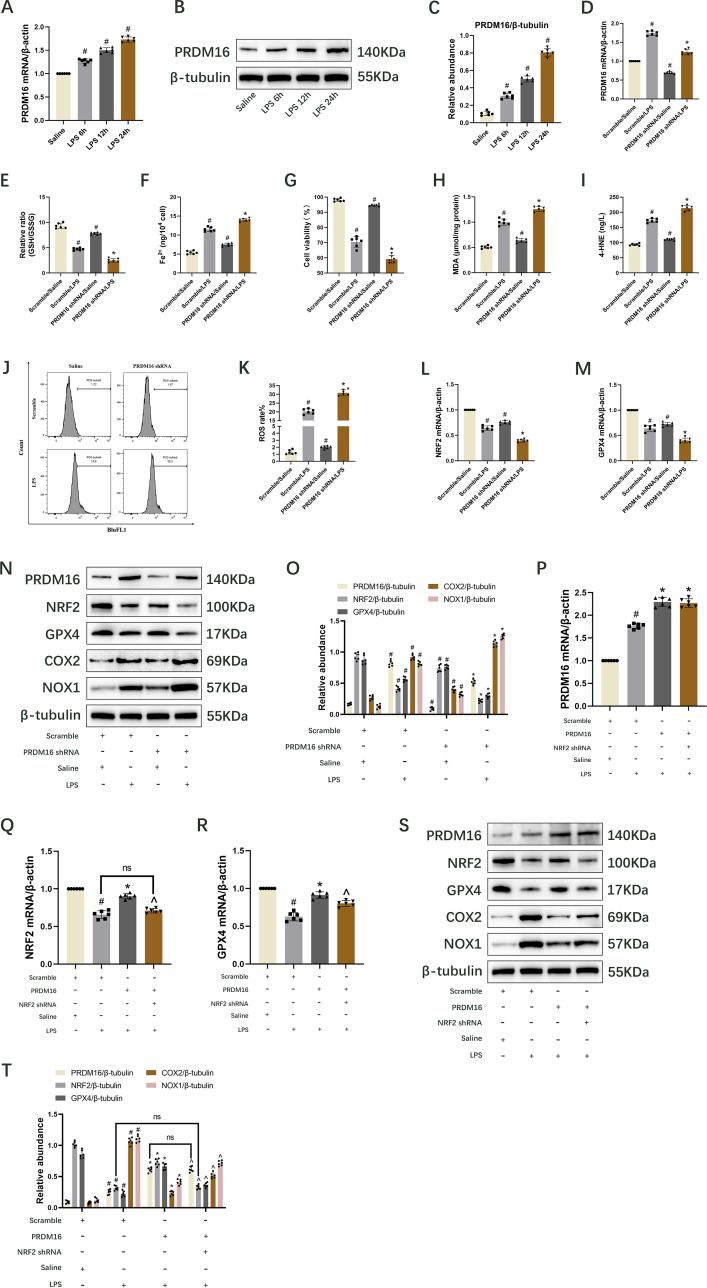


**figs4:**
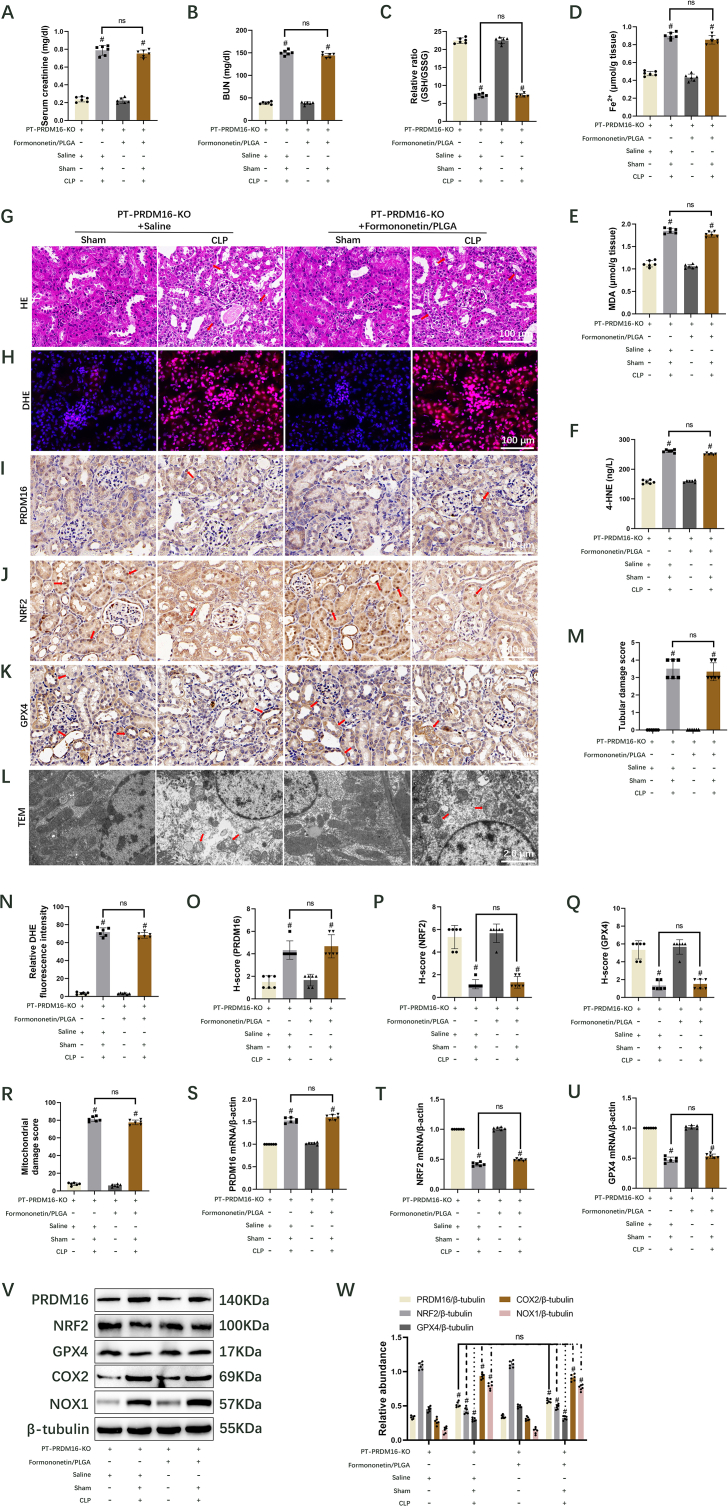


**figs5:**
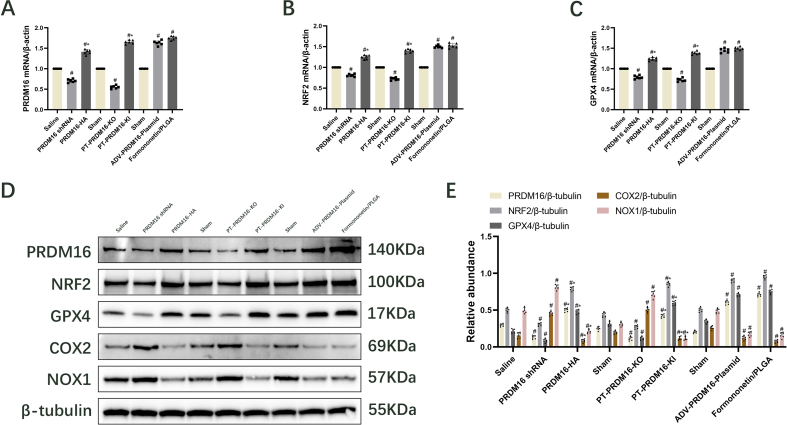


**figs6:**