# Supplementary material for: PRDM16 suppresses ferroptosis to protect against sepsis-associated acute kidney injury by targeting the NRF2/GPX4 axis
Source: Redox Biol. 2024 Nov 7;78:103417. doi: 10.1016/j.redox.2024.103417 (PMC11612791; doi:10.1016/j.redox.2024.103417)
Supplement: Multimedia component 1 [file mmc1.docx]

**Supplementary Figure legends**

**Fig. 1.** Mean ± SD (n = 6). (A) Immunohistochemical staining of PRDM16 in the outer medulla. The nuclei stained positive for PRDM16 were indicated by the arrows. Scale bar: 100 µm. (B) Immunohistochemical staining of PRDM16 in the inner medulla. The nuclei stained positive for PRDM16 were indicated by the arrows. Scale bar: 100 µm. (C and D) H-score for PRDM16. ^#^ indicates significance versus Sham.

**Fig. 2.** Mean ± SD (n = 6). (A, D, G, J, and M) Cell viability (%). (B, E, H, K, N, P, and R) MDA levels. (C, F, I, L, O, Q, and S) 4-HNE concentrations. ^#^ indicates significance versus Scrambled/Saline and PT-PRDM16-WT/Sham cohorts, respectively. ^*^ indicates significance compared to Scrambled/LPS and PT-PRDM16-WT/CLP cohorts, respectively. ^^^ indicates significance versus Scrambled/LPS, PRDM16 shRNA/LPS, PRDM16-HA/LPS cohorts, respectively. ^&^ indicates significance versus Scrambled/LPS, PRDM16 shRNA/LPS, PRDM16-HA/LPS, Fer-1/LPS, and Erastin/LPS cohorts, respectively.

**Fig. 3.** PRDM16 facilitates ferroptosis resistance against sepsis-induced damage in HK-2 cells by targeting the NRF2/GPX4 or GPX4 pathway. HK-2 cells were treated with LPS for 6, 12, and 24 h. Following transfection with PRDM16 shRNA, PRDM16 plasmid, or scrambled sequence, cells were subjected to LPS exposure for 24 h. Data are presented as Mean ± SD (n = 6). (A) PRDM16 expression quantified by RT-qPCR. (B) Levels of PRDM16 and β-tubulin determined by immunoblotting. (C) Quantification of immunoblotting results. (D) PRDM16 expression assessed by RT-qPCR. (E) GSH/GSSG ratio. (F) Levels of Fe^2+^ expression. (G) Cell viability percentage. (H) MDA levels. (I) 4-HNE concentrations. (J) ROS levels in cells, determined by FCM. (K) ROS rate analysis. (L and M) NRF2 and GPX4 expression measured by RT-qPCR. (N) Levels of PRDM16, NRF2, GPX4, COX2, NOX1, and β-tubulin were assessed by immunoblotting. (O) Immunoblot quantification. (P-R) RT–qPCR quantification of PRDM16, NRF2, and GPX4. (S) Immunoblotting of PRDM16, NRF2, GPX4, COX2, NOX1, and β-tubulin. (T) Quantification of immunoblotting results. ^#^, ^*^ indicate significance compared to Scrambled/Saline and /LPS cohorts, respectively. ^ denotes significance versus Scrambled/LPS and PRDM16/LPS cohorts, respectively.

**Fig. 4.** The protective effect of Formononetin/PLGA failed to function in the CLP model of PT-PRDM16-KO mice. Mouse models of CLP were established, and blood samples and kidney tissues were collected 18 h post-CLP. Data are presented as Mean ± SD (n = 6). (A) Serum creatinine. (B) BUN levels. (C) GSH/GSSG ratio. (D) Levels of Fe^2+^ expression. (E) MDA levels. (F) 4-HNE concentrations. (G) H&E staining (Scale bar: 100 µm). The damaged renal tubules were indicated by the arrows. (H) DHE staining (Scale bar: 100 µm). (I) Immunohistochemical staining of PRDM16 (Scale bar: 100 µm). The nuclei stained positive for PRDM16 were indicated by the arrows. (J) Immunohistochemical staining of NRF2 (Scale bar: 100 µm). The nuclei stained positive for NRF2 were indicated by the arrows. (K) Immunohistochemical staining of GPX4 (Scale bar: 100 µm). The renal tubules stained positive for GPX4 were indicated by the arrows. (L) TEM images of the renal cortex. (Scale bar: 2.0 µm). The damaged mitochondria were indicated by the arrows. (M) Tubular damage score. (N) Relative DHE fluorescence intensity. (O) H-score for PRDM16. (P) H-score for NRF2. (Q) H-score for GPX4. (R) Mitochondrial damage score. (S-U) RT–qPCR quantification of PRDM16, NRF2, and GPX4. (V) Immunoblotting of PRDM16, NRF2, GPX4, COX2, NOX1, and β-tubulin. (W) Quantification of immunoblotting results. ^#^ indicate significance versus PT-PRDM16-KO/Sham and /Formononetin/PLGA cohorts, respectively.

**Fig. 5.** PRDM16 binds to the promoter regions of NRF2 or GPX4, enhancing their expression and ultimately promoting the expression of GPX4 under basal conditions. Data are presented as Mean ± SD (n = 6). (A-C) PRDM16, NRF2, and GPX4 expression quantified by RT-qPCR. (D) Immunoblotting of PRDM16, NRF2, GPX4, COX2, NOX1, and β-tubulin. (E) Quantification of immunoblotting results. ^#^ denotes significance compared to Saline or Sham cohorts. ^*^ indicates significance compared to PRDM16 shRNA or PT-PRDM16-KO cohorts.
